# The challenge of balancing model sensitivity and robustness in predicting yields: a benchmarking study of amide coupling reactions†

**DOI:** 10.1039/d3sc03902a

**Published:** 2023-09-13

**Authors:** Zhen Liu, Yurii S. Moroz, Olexandr Isayev

**Affiliations:** a Department of Chemistry, Mellon College of Science, Carnegie Mellon University Pittsburgh PA 15213 USA olexandr@olexandrisayev.com; b Enamine Ltd Kyïv 02660 Ukraine; c Chemspace LLC Kyïv 02094 Ukraine; d Taras Shevchenko National University of Kyïv Kyïv 01601 Ukraine

## Abstract

Accurate prediction of reaction yield is the holy grail for computer-assisted synthesis prediction, but current models have failed to generalize to large literature datasets. To understand the causes and inspire future design, we systematically benchmarked the yield prediction task. We carefully curated and augmented a literature dataset of 41 239 amide coupling reactions, each with information on reactants, products, intermediates, yields, and reaction contexts, and provided 3D structures for the molecules. We calculated molecular features related to 2D and 3D structure information, as well as physical and electronic properties. These descriptors were paired with 4 categories of machine learning methods (linear, kernel, ensemble, and neural network), yielding valuable benchmarks about feature and model performance. Despite the excellent performance on a high-throughput experiment (HTE) dataset (*R*^2^ around 0.9), no method gave satisfactory results on the literature data. The best performance was an *R*^2^ of 0.395 ± 0.020 using the stack technique. Error analysis revealed that *reactivity cliff* and *yield uncertainty* are among the main reasons for incorrect predictions. Removing reactivity cliffs and uncertain reactions boosted the *R*^2^ to 0.457 ± 0.006. These results highlight that yield prediction models must be sensitive to the reactivity change due to the subtle structure variance, as well as be robust to the uncertainty associated with yield measurements.

## Abstract 2

A sensitive model captures the reactivity cliffs but overfit to yield outliers. On the other hand, a robust model disregards the yield outliers but underfits the reactivity cliffs.

## Graphical abstract

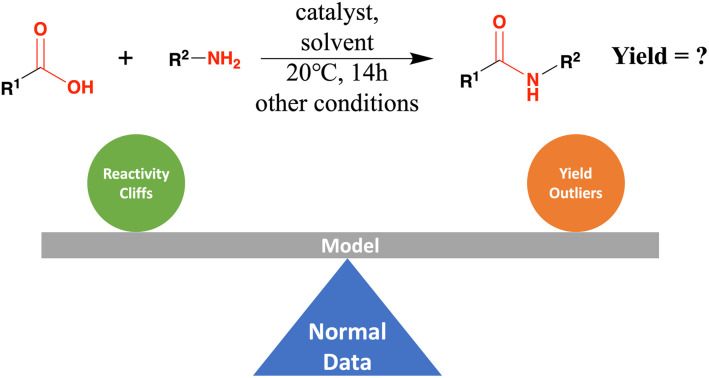

## Introduction

Computer-assisted synthesis prediction (CASP) is a field of computational chemistry that aims to develop algorithms and software tools to assist chemists in predicting the outcomes of chemical reactions. CASP uses machine learning (ML) and artificial intelligence (AI) techniques to predict the feasibility, yield, and optimal conditions for a chemical reaction. Recent exploratory studies in the field of reaction predictions, show applications in retrosynthesis,^1,2^ product prediction,^3–5^ selectivity,^6^ and other relevant tasks.^7,8^ Accurately predicting reaction yields is one of the key objectives in CASP as many reaction-related tasks can be framed as yield optimization problems. Yield serves as the ultimate metric for selecting reagents in a single reaction or planning a synthesis pathway. However, despite its importance, predicting the theoretical yield remains challenging because the yield depends on many observable and unobservable factors throughout the reaction process, including the interaction between molecules, environment conditions, and human operations.

While impressive yield prediction performance (*R*^2^ is around 0.9) has been achieved in many high-throughput experiment (HTE) datasets, the yield prediction *R*^2^ score on large literature datasets is usually unsatisfactory.^9–16^ For example, the Doyle group reported an example of predicting reaction yields with a random forest model on the Buchwald–Hartwig HTE dataset.^9^ The dataset contains 4608 C–N cross-coupling reactions and the *R*^2^ score and mean absolute error (MAE) were 0.92 and 7.8%, respectively. Since then, the dataset has become a standard benchmark dataset for many yield prediction models. Schwaller *et al.* reported a Yield-BERT model for reaction yield predictions.^10^ Although the *R*^2^ score for the yield prediction task was as high as 0.94 on the Buchwald–Hartwig dataset,^9^ the performance dropped sharply (*i.e.*, *R*^2^ around 0.2) on the literature dataset.^17,18^ The staggering performance difference of yield prediction on the HTE dataset and the literature dataset is widespread. Recently, Grzybowski^11^ and Glorius^15^ studied this phenomenon, suggesting that the unsatisfactory ML performance may be due to the popular trend in the literature dataset induced by human bias in experiment design and result reporting. However, augmenting the dataset with zero or low-yield reactions did not significantly improve the performance, indicating that additional factors might degrade the model performance.

To understand the causes for failures in a large literature dataset, we systematically investigated the yield prediction task. We tested 4 categories of ML models (*i.e.*, linear methods, kernel methods, ensemble methods, and neural networks) on an HTE yield dataset and a large literature yield dataset. We utilized a set of 4608 Buchwald–Hartwig reactions from Doyle^9^*et al.* to represent the HTE dataset, given its extensive prior modeling. We curated 41 239 amide coupling reactions from Reaxys^19^ to represent the literature dataset. These reactions were chosen due to their significance in medicinal chemistry and the substantial volume of available data. While the Buchwald–Hartwig reactions and the amide coupling reactions are very different, they possess characteristics inherent to the HTE and large literature datasets, respectively. The phenomena observed in the context of Buchwald–Hartwig reactions and amide coupling reactions can be extrapolated to typical HTE datasets and large literature datasets, respectively. Besides the SMILES of reactants and products, the reaction context (*i.e.*, time, temperature, reagents, condition, and solvent) was also extracted where possible from Reaxys to construct the amide coupling dataset. Please note that the reaction yields were extracted as they appeared in the Reaxys database, regardless of the reaction scale. Also, we augmented the literature dataset with reaction intermediates, optimized 3D structures of the molecules, and 2D/3D descriptors derived from the SMILES and conformers. All amide coupling reactions were catalyzed by carbodiimides to minimize irrelevant variables in this investigation. The carbodiimides include 1-ethyl-3-(3-dimethylaminopropyl)carbodiimide (EDC), *N*,*N*′-dicyclohexylcarbodiimide (DCC), and *N*,*N*′-diisopropylcarbodiimide (DIC). The combination of different reaction descriptors and model categories enabled a systematic yield prediction benchmark, providing insights into the key factors that influence the reaction yield prediction challenge.

Our results demonstrated that most models gave unsatisfactory predictions (*R*^2^ < 0.5) in a large and diverse literature dataset even if they achieved excellent predictions (*R*^2^ > 0.9) on a carefully curated HTE dataset. This highlights that a large real-world reaction dataset is necessary to evaluate the model capacities. Moreover, incorporating the reaction context (*i.e.*, solvent, temperature, *etc.*) generally improves the model performance. By taking the average prediction from multimodal information (*i.e.*, descriptors that contain information in different aspects), we improved the performance on the literature amide coupling dataset, where the *R*^2^ and MAE are 0.395 ± 0.020 and 13.42% ± 0.25%, respectively. Lastly, we investigated the reactions where the model made significant incorrect predictions and found that the reactivity cliffs and uncertain reaction records played a key role in degrading the model performance. The yield discrepancies in the reaction dataset exemplify the complexity of the structure–yield relationship and the intrinsic uncertainty of reaction yields, highlighting the importance of robust ML methods that can detect correct signals among noisy labels.

## Results and discussion

### The amide coupling dataset

**Fig. 1:**
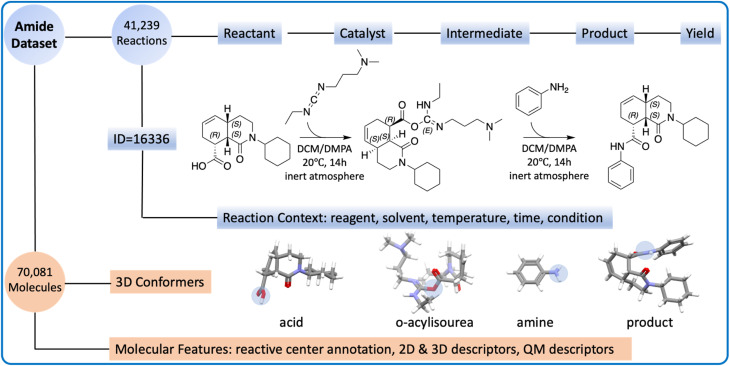
The overview of the amide coupling dataset. The reaction part (highlighted in blue) contains SMILES and context for the reactions. The reaction (ID = 16336) is visualized, where the carboxylic acid first reacts with EDC to form an intermediate (*O*-acylisourea). The amine serves as the nucleophile, which attacks the intermediate and forms the product. The molecule part (highlighted in orange) contains molecular information and descriptors. For the 3D conformer, the reactive center is highlighted in the blue circle.

All reactions strictly adhere to the pattern of “A + B = C + H_2_O”, where A, B, and C are carboxylic acid, amine, and the product, respectively. All reactions are catalyzed by carbodiimides, following the same reaction mechanism (Fig. S1†). The amide coupling dataset consists of two components: the *reaction part* and the *molecule part* (Fig. 1).

The *reaction part* is represented as a CSV file. It contains 41 239 reaction records, each of which lists the reaction ID, reactant SMILES, product SMILES, yield, and context. The reaction context includes the reagent, solvent, time, temperature, and other relevant conditions, though not all reactions have the complete reaction context information. The reagents are sometimes mixed with catalysts or solvents because the definition of a reagent is ambiguous.

The *molecule part* contains optimized low-energy 3D conformers and molecular descriptors. Combining carboxylic acids, amines, products, and *O*-acylisoureas, there are 70 081 unique molecules in the amide coupling dataset. The low-energy conformer for each molecule was generated with Auto3D,^20^ where the isomerization engine was Omega^21^ and the optimization engine was AIMNET.^22^ The reactive centers (*i.e.*, atoms whose connectivity changed during the reaction) were annotated using Algorithm 1 as shown in the ESI.† From SMILES and conformers, we derived 4 descriptors: Morgan fingerprints, Mordred features, atomic environment vectors (AEV), and QM features. More details about the preparation of the dataset can be found in the Method section.

**Fig. 2:**
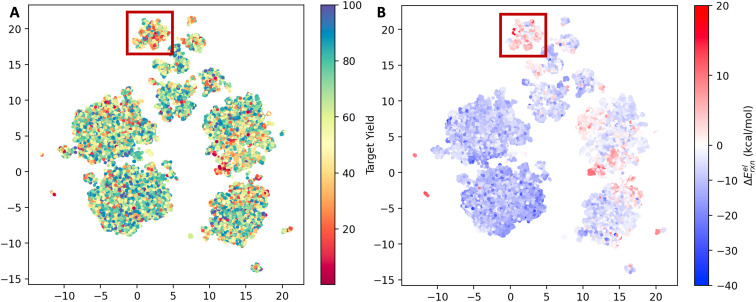
UMAP projection of the reaction space. (A) Points color-coded by experimental yield (%). (B) Points color-coded by the computed electronic reaction energy Δ*E*^el^_rxn_.

The reaction space was visualized with UMAP^23^ using the AIMNET embedding of the reaction centers (Fig. 2). AIMNET embedding captures the local environment of the reaction centers.^22^ The plot demonstrates the diversity within the chemical space. The AIMNET embedding was not specifically trained for yields, hence the mixed distribution of yields in panel A. Additionally, some of our QM features showed a weak correlation with the reaction yield, for example, the electronic reaction energy Δ*E*^el^_rxn_. It is defined as follows,Δ*E*^el^_rxn_ = *E*^el^_product_ + *E*^el^_water_ − *E*^el^_acid_ − *E*^el^_amine_

For most reactions, Δ*E*^el^_rxn_ tends to be negative. Comparing panel B and panel A, we observed that regions with a concentration of low-yield reactions often exhibit high electronic reaction energy (indicated by the red box). Additional visualization can be found in Fig. S3.†

### Yield prediction on the amide coupling dataset

We applied 4 categories of ML models (*i.e.*, linear methods, kernel methods, ensemble methods, and neural networks) for reaction yield prediction. Each method was supplied with appropriate input formats, resulting in 14 benchmark combinations (Table 1). Overall, none of the methods gave satisfactory results. The baseline is a dummy model that always outputs the mean value (64.1%) of the yields in the dataset. The best result came from RF using fingerprints as the input, where the *R*^2^ and MAE values were 0.378 and 13.50%, respectively.

**Table 1:** Yield prediction using 2D features on the amide coupling dataset

Category	Model	Features	*R* ^2^	MAE (%)
Baseline	Mean	N/A	0.00 ± 0.00	18.46 ± 0.24
Linear methods	Ridge	Mordred descriptors	0.182 ± 0.029	16.02 ± 0.15
		Morgan fingerprints	0.181 ± 0.015	16.03 ± 0.11
	Lasso	Mordred descriptors	0.135 ± 0.015	17.01 ± 0.17
		Morgan fingerprints	0.00 ± 0.00	18.46 ± 0.27
Kernel methods	SVM	Mordred descriptors	−0.01 ± 0.00	18.29 ± 0.28
		Morgan fingerprints	0.028 ± 0.013	18.80 ± 0.19
Ensemble methods	RF	Mordred descriptors	0.345 ± 0.010	14.25 ± 0.12
		Morgan fingerprints	**0.378 ± 0.009**	**13.50 ± 0.13**
	GBM	Mordred descriptors	0.326 ± 0.014	14.27 ± 0.03
		Morgan fingerprints	0.350 ± 0.010	13.95 ± 0.19
Neural networks	Yield-BERT^10^	Reaction SMILES	0.181 ± 0.017	16.26 ± 0.18
	NNConv^24^	2D reaction graph	0.130 ± 0.048	16.77 ± 0.88
	MFConv^25^	2D reaction graph	0.200 ± 0.013	14.60 ± 0.94
	AttentiveFP^26^	2D reaction graph	0.130 ± 0.027	16.09 ± 0.31

Ensemble methods gave significantly better results than the other three types of models (linear methods, kernel methods, and neural networks). In terms of *R*^2^ values, the ensemble method was around 0.35 while other methods could hardly go beyond 0.20. NNConv, MFConv and AttentiveFP achieved impressive performance on many quantitative structure property relationship (QSPR) experiments,^24–26^ yet the *R*^2^ scores for yield prediction were 0.130, 0.200 and 0.130, respectively. Yield-BERT is a well-known model for reaction yield prediction, but its *R*^2^ score was only 0.181. Besides giving better yield prediction performance, ensemble methods are also robust. Changing model hyperparameters for ensemble models usually did not change the results much, but other methods were extremely sensitive to the selection of model hyperparameters. The advantages of ensemble models come from their design: training several base predictors at the same time and then combining their predictions at the test time. This improves the model generalizability and robustness. There was no obvious winner descriptor that gave significantly better performance than others, though the combination of Morgan fingerprint and RF had a slightly higher *R*^2^ score than the others.

### Yield prediction on the Buchwald–Hartwig dataset

The results on the amide coupling dataset drove us to test the models using a control experiment. The goal is to understand whether the previous unsatisfactory results were due to the inability of the models or the complexity of the amide coupling dataset. The Buchwald–Hartwig (BH) dataset was used for the yield prediction control experiment. It is an HTE dataset commonly used to evaluate model performance for yield prediction tasks.^10,13,27^ As for the models, we still used 4 categories: linear methods, kernel methods, ensemble methods, and neural networks. To be consistent with the original paper,^9^ the following results were obtained with 5 random train-test-splits where each training and testing set contains 70% and 30% of the dataset, respectively.

The accuracy on the Buchwald–Hartwig dataset was generally satisfactory (Table 2). The best *R*^2^ score and MAE value, which came from Yield-BERT, were 0.934 and 4.60%, respectively. Several other models (SVM and RF) also achieved *R*^2^ values that were larger than 0.90. The most common *R*^2^ score was around 0.65. Even the lowest *R*^2^ score was 0.474, which is higher than the best *R*^2^ score on the amide coupling dataset.

**Table 2:** Yield prediction using 2D features for the Buchwald–Hartwig dataset

Category	Model	Features	*R* ^2^	MAE (%)
Baseline	Mean	N/A	0.00 ± 0.00	23.51 ± 0.22
Linear methods	Least squares	Mordred descriptors	0.688 ± 0.012	12.27 ± 0.18
		Morgan fingerprints	0.662 ± 0.017	12.77 ± 0.25
Kernel methods	SVM	Mordred descriptors	0.474 ± 0.023	14.77 ± 0.22
		Morgan fingerprints	0.906 ± 0.005	6.08 ± 0.18
Ensemble methods	Random forest	Mordred descriptors	0.920 ± 0.006	5.22 ± 0.11
		Morgan fingerprints	0.922 ± 0.006	5.18 ± 0.11
Neural networks	Yield-BERT^10^	Reaction SMILES	**0.934 ± 0.007**	**4.60 ± 0.21**
	NNConv^24^	2D reaction graph	0.650 ± 0.012	10.28 ± 0.17
	MFConv^25^	2D reaction graph	0.602 ± 0.031	12.08 ± 0.87
	AttentiveFP^26^	2D reaction graph	0.648 ± 0.036	10.63 ± 0.75

### Comparing model performances on the amide coupling dataset and the BH dataset

**Fig. 3:**
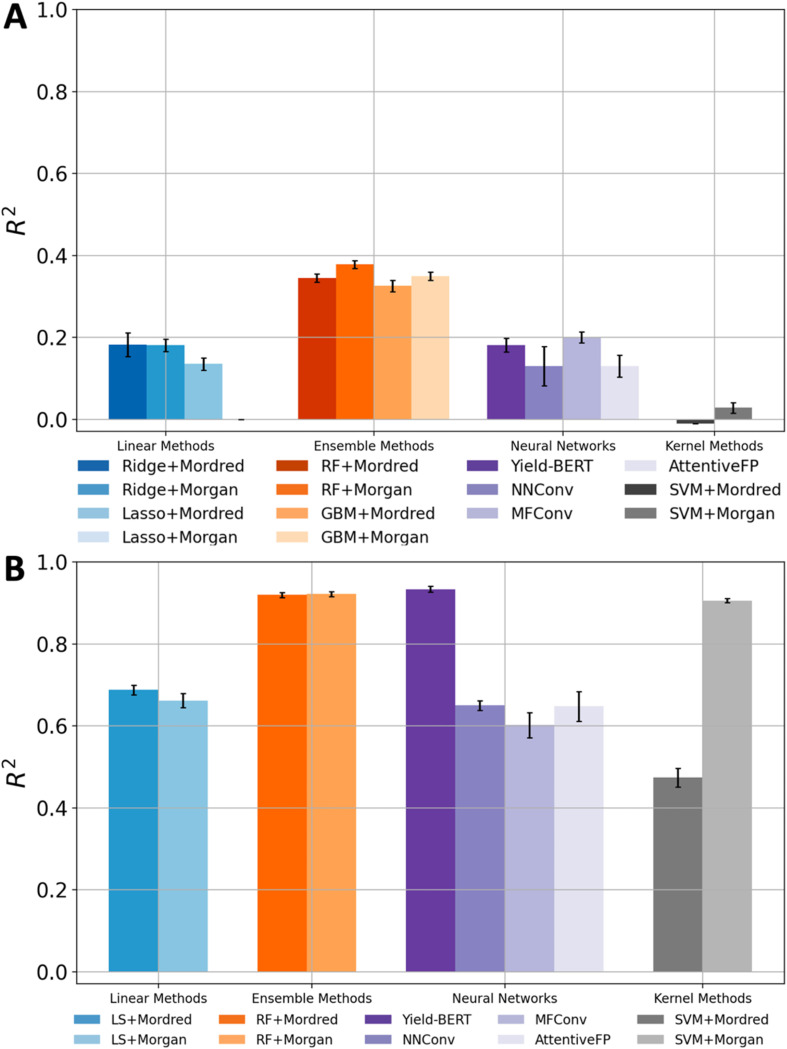
Summary of *R*^2^ scores on the amide coupling dataset (A) and the Buchwald–Hartwig dataset (B).

All methods demonstrated a significant improvement in yield prediction accuracy on the BH dataset compared with their performance on the amide coupling dataset (Fig. 3). Notably, even the worst models in the previous section delivered good or excellent prediction accuracy. For example, the linear and SVM methods, which both gave *R*^2^ values of around 0 on the amide coupling dataset, now achieved an *R*^2^ as high as 0.668 and 0.906, respectively. The GNNs only achieved moderate accuracies, which may be due to the limited amount of data. The GNN contains a lot of parameters, which generally require a large training dataset. However, our Buchwald–Hartwig dataset contains only around 4.5 thousand reactions. The exceptional performance of Yield-BERT is probably because it is based on a pre-trained language model. RF is again robust and usually gives an *R*^2^ of around 0.92. A similar trend was observed when using MAE as the evaluation metric (Fig. S6†).

Since the models can give accurate yield prediction on the BH dataset, the previous unsatisfactory yield prediction accuracy on the amide coupling dataset is likely a result of the data complexity. So far, all methods only used information derived from reactant and product SMILES. This was the practical choice for most yield prediction models due to the limited availability of comprehensive reaction information. Recent tools enabled us to gather more reaction information, such as 3D structures and QM descriptors.^20,22,28^

### Incorporating additional information

We considered the following additional information: reaction context, 3D information, and QM descriptors. The reaction context means the time, temperature, reagent, solvent, catalyst, and other available information. The reaction context is informative because it influences the interaction between reactants, and consequently the final reaction yield. For each reaction, the context was embedded as a one-hot vector of length 735. As for the 3D information, we used AEV and steric descriptors. AEV has been shown to be an effective method for encoding conformers in several applications.^22,29^ The smooth overlap of atomic positions (SOAP) descriptor^30^ was also used in comparison with the AEV descriptor. Both descriptors describe information about the spatial environment of each atom. The steric descriptors^31^ describe the buried volume of reaction centers. They could implicitly represent the steric effect, which is one of the dominant factors in S_N_2 reactions. The amide coupling reaction is an example of an S_N_2 reaction. The QM descriptors quantitatively capture molecular and reactivity characteristics. For example, the Fukui index of a carboxylic acid describes how likely it is to accept an extra electron. These characteristics have been proven to be useful for reaction-related predictions.^9,14,32^ Our QM calculator, the AIMNET-NSE model,^22^ only applies to molecules consisting of H, C, N, O, F, Si, P, S, and Cl. These elements make up 38 157 reactions in our dataset. The following experiments were carried out with these reactions.

As summarized in the first 5 rows of Table 3, applying the 3D descriptor alone did not improve the yield prediction performance. Because RF usually outperforms other methods and is not very sensitive to the selection of hyperparameters, we exploited RF to study the effects of combining different descriptors. Combining the context embedding with either 2D or 3D information slightly improves the yield prediction performance. Surprisingly, the QM descriptor did not help with the yield prediction task. The combination of fingerprint and reaction context was the most powerful reaction descriptor, where the *R*^2^ and MAE are 0.389 and 13.30%, respectively.

**Table 3:** Yield prediction using additional information

Model	Features	*R* ^2^	MAE (%)
RF	AEV	0.362 ± 0.008	13.95 ± 0.09
RF	SOAP^a^	0.307 ± 0.010	15.05 ± 0.13
GBM	AEV	0.337 ± 0.009	14.08 ± 0.13
MLP	AEV	0.284 ± 0.010	14.18 ± 0.07
MLP	Steric descriptor	0.182 ± 0.041	15.69 ± 0.40
RF	AEV + context	0.367 ± 0.007	13.87 ± 0.09
	FP + context	**0.389 ± 0.010**	**13.30 ± 0.13**
	QM descriptors^a^	0.245 ± 0.008	15.76 ± 0.19
	QM + context^a^	0.281 ± 0.012	15.34 ± 0.18
	AEV + QM descriptors^a^	0.369 ± 0.015	14.07 ± 0.20
	FP + QM descriptors^a^	0.363 ± 0.016	14.11 ± 0.17

a Due to the availability of QM descriptors, only 38 157 reactions were used for consistency.

Each category of descriptor describes different aspects of the reaction, making them multimodal information sources. To mitigate the “curse of dimension” while combining all available descriptors, recursive feature elimination (RFE) was applied to select the most informative features in each descriptor category (see the ESI† for details). This ended up with a merged descriptor with a length of 2705 (324 fingerprint features, 51 QM features, 1295 Mordred features, and 1035 AEV features). The *R*^2^ value dropped to 0.338 when we used RF on the combined features, suggesting that RF is not optimal for learning from multimodal information.

**Fig. 4:**
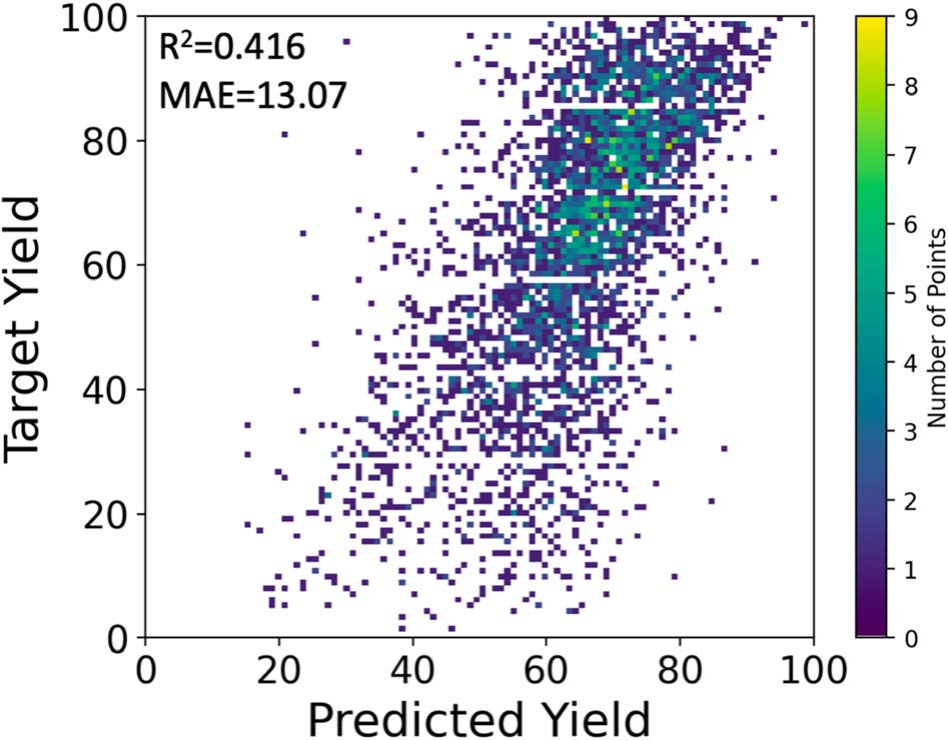
The target and predicted yields (%) by stacking MLPs.

We used the stacking technique to improve the prediction performance using multiple descriptors. First, we trained four base models, one for each descriptor, which were relatively weak on their own. The base models consisted of multilayer perceptron (MLP) or random forest (RF) models. Then, we used a meta-model to combine the predictions of the four base models and make a final prediction. In our case, the meta-model outputs the average of the predictions from the four base models. Although MLP showed lower performance than RF when using single descriptors, the average prediction from the four individual MLP models was better than that of RFs, resulting in a higher *R*^2^ score of 0.395 ± 0.020 for MLP compared to 0.363 ± 0.014 for RF. The stacking approach improved the peak performance of the MLP model, achieving an *R*^2^ of 0.416 and MAE of 13.07%, as shown in Fig. 4.

We also investigated the impact of amine types on the reaction yields. The amide coupling reaction dataset was divided into 3 subsets based on the amine types: primary aliphatic amine, primary aromatic amine and secondary amine. We trained and evaluated a model for each subset. The *R*^2^ values were as high as 0.425 ± 0.015 and 0.424 ± 0.07 for the primary aromatic amine subset and secondary amine subset, respectively. However, the *R*^2^ value was just 0.363 ± 0.004 for the primary aliphatic amine subset (Table S3†).

For yield prediction models to be useful for guiding reaction planning in real life, this performance needs to be improved significantly. Grzybowski^33^ and Glorius^15^ pointed out that the unsatisfactory results may be due to the imbalanced distribution of the dataset. For reaction yield prediction, this refers to the concentration of high-yield reactions in the literature dataset as a result of human bias in experiment design and result reporting. However, we did not observe a significant increase in terms of yield prediction accuracy after injecting artificial negative reactions into the training set (see Table S4†), which is consistent with the results obtained by Glorius^15^*et al.* This led us to the question, are there any other factors that compromise the yield prediction performance?

### The challenge of balancing sensitivity and robustness

**Fig. 5:**
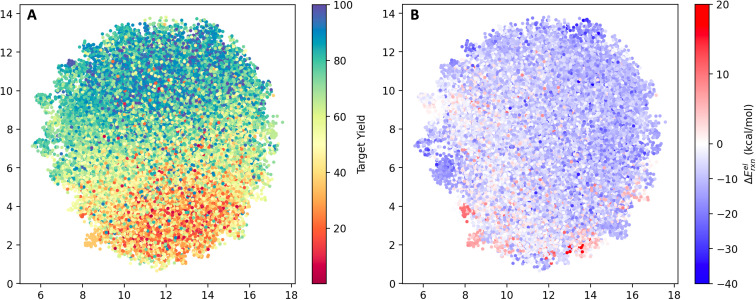
UMAP of the reaction space in the view of the model. (A) Points color-coded by experimental yield (%). (B) Points color-coded by computed electronic reaction energy Δ*E*^el^_rxn_.

The model's view of the training and testing datasets was visualized using UMAP with the last layer embedding (Fig. 5). Following the training process, the model successfully learned the distribution of yields in latent space. In panel A, we observed an increase in yield from the bottom (red) to the top (blue) part of the plot. However, there are outliers. For instance, a few blue dots are located within the red region, and some red dots can be found within the blue region. These outliers indicate the presence of noise within the dataset. By comparing panel B and panel A, we observed a subtle clustering between low-yield regions and high electronic reaction energies, as well as high-yield regions and low electronic reaction energies. The Pearson correlation coefficient between the yield and the electronic reaction energy Δ*E*^el^_rxn_ is −0.12. As a feature in the QM descriptor, a reasonable relationship with the target variable is useful.

**Fig. 6:**
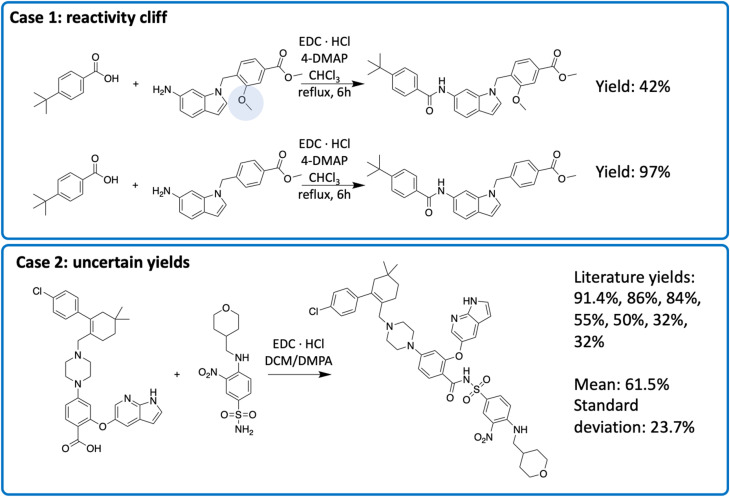
Two types of uncertainty in the reaction yields. The exact conditions may be different in multiple sources, though the reactant, solvent and catalyst are the same.

To understand the source of errors, we examined reactions with large prediction errors. The absolute prediction error ranged from 59.0 to 68.7, indicating that the model predictions and the actual yields were almost completely opposite. Half of these errors were attributed to reactivity cliffs or “uncertain” data points (Fig. 6). Reactivity cliffs are characterized by reactions with highly similar reactants and context, indicated by a cosine similarity greater than 0.9, yet the yield difference is at least 30%. We identified 6365 reactivity cliffs in the amide coupling dataset. For example, in case 1 in Fig. 6, the top reaction only contains an extra methoxy group (highlighted in blue) that is far from the reaction center, but the yield is 55.0% lower than that of the bottom reaction.^34^ Reactivity cliffs highlight that a small change in the structure could lead to a significant change in the reactivity, akin to the reactivity cliff observed elsewhere.^35^ Uncertain data points refer to the reactions with multiple different yield records. For example, for the reaction in case 2 in Fig. 6 (the final step in the synthesis of Venetoclax), the yields range from 32.0% to 91.4% in 7 different literature sources.^36–42^ We identified 649 uncertain data points in the amide coupling dataset. This uncertainty does not necessarily imply that experimental results are inaccurate, but rather stems from the fact that the yield of a reaction is influenced by a multitude of factors, such as the nature of the molecules, environmental conditions, and operational differences, which can introduce stochasticity in the measurement. The process of identifying reactivity cliffs and uncertain reactions is detailed in the ESI,† with additional examples.

To address the reactivity cliffs, a model must exhibit sensitivity to yield changes resulting from subtle structural variations. However, the model must also demonstrate robustness against yield uncertainties arising from measurement variances. A sensitive model captures the reactivity cliffs but overfits to yield outliers. On the other hand, a robust model disregards the yield outliers but underfits the reactivity cliffs. In both cases, the overall performance of the model suffers. This presents a distinctive challenge for the task of predicting reaction yields, as the model needs to strike a balance between these two conflicting requirements. Reactivity cliffs and uncertain reactions are prevalent throughout large reaction datasets, making it difficult for the model to learn a meaningful mapping from the feature space to the label space.

Compared with the HTE dataset, the amide dataset contains around 10 times more reactions, around 1500 times more unique molecules, and comprehensive (though not complete) information about molecules and reactions. We could identify many similar reactions and observe how a subtle structure change could lead to the difference in the final yield. This provides the opportunity to observe the reactivity cliffs and uncertain yields. The HTE dataset also contains reactivity cliffs, but the number of unique molecules is so small that the model could simply remember the reactivity cliffs instead of learning the reactivity cliff from structures. For example, some reactants tend to have better reactivity than others in a HTE dataset, and the model could trivially predict high yields for reactions with these reactants. In contrast, there is no way for a model to cheat on the amide dataset due to the large number of unique molecules. The model must learn the reactivity cliffs. The amide dataset also provides the condition for testing model robustness to yield uncertainty, because the reactions are reported from different resources. A side-by-side comparison between the HTE dataset and the amide dataset can be found in Table S5.†

After removing the above 6365 reactivity cliffs and 649 uncertain data points, the stacking model performance significantly improved, where the *R*^2^ and MAE were 0.457 ± 0.006 and 12.31% ± 0.16%, respectively. This is the best performance for an amide coupling yield prediction task on a large literature dataset so far,^10,11,15^ though this performance is subject to dataset selection, train-test-splits and other factors. Our result suggested that the reactivity cliffs and uncertain data are among the major factors that compromise the model performance. These reactivity cliffs or uncertain data points pose unique challenges for predicting reaction yields. On the one hand, the model must exhibit sufficient sensitivity to account for the impact of subtle structural changes on yields. On the other hand, it must also accommodate the inherent uncertainty associated with yield measurements. Our case study offers only a limited glimpse into the magnitude of inherent uncertainty within the reaction yield dataset. Examining each reaction manually within the dataset is an impractical endeavor, compounded by the lack of a precise definition for reactivity cliffs and uncertain reactions. The task of curating a dataset of superior quality presents its own challenges. Moreover, the current *R*^2^ score of 0.457 falls notably short of being satisfactory, implying the possible existence of additional factors that undermine the accuracy of yield predictions.^15,33^

## Conclusions

Reaction yield prediction is a very important yet unsolved task. Most reaction-related prediction tasks can be reformulated as yield optimization tasks; however, current models fail on yield prediction for large literature datasets. We provided a systematic benchmark through a variate combination of ML models and descriptors. Our results revealed that current models can handle high-throughput experimental data but encounter difficulties when dealing with complex literature datasets. Despite this, by merging information from multimodal descriptors, we achieved the best performance for yield prediction on a large literature dataset. The results highlight the benefits of combining information from multiple types of descriptors to model complex chemical properties. These findings will offer valuable insights for guiding the model selection and informing descriptor design in future research endeavors.

We observed that reactivity cliffs and uncertain yields severely degraded model performance. The reactivity cliffs and uncertain yields highlight the complexity of the structure–yield relationship and the reaction reproducibility issue, respectively. It is challenging for a model to be both sensitive to reactivity change caused by subtle structure variance, as well as be robust to uncertainties in yield measurements.

During our investigation, we constructed a dataset of 41 239 amide coupling reactions containing comprehensive information about reactants, intermediates, products, context, and yield. Optimized 3D structures were provided for the molecules. Based on the SMILES and 3D structures, we derived 2D and 3D descriptors for the reactions. The methods for preparing molecular descriptors are generally applicable to other reactions, and this dataset presents a challenging benchmark for yield prediction that supports various machine learning models.

Our findings underscore the importance of high-quality reaction datasets and the necessity to address uncertainties in target variables. While significant challenges remain, we also recognize numerous opportunities for advancement. The emergence of cloud labs and lab automation holds promise for the creation of large-scale, reproducible reaction datasets. As an alternative task, predicting the conversion rate could prove valuable. Lastly, the rapid progress in AI technology may provide effective methods to learn correct signals from noisy labels.

## Methods

### Processing Reaxys reactions

The reaction part was augmented and improved in the following ways. Firstly, the SMILES of individual molecules were extracted from the reaction SMILES. The raw dataset only contained reaction SMILES, from which the SMILES for carboxylic acid, amine, and the product were extracted. Secondly, a unique reaction ID was assigned to each unique reaction in the dataset. A reaction is considered unique if the reactants, product, or context is different from those of the remaining reactions. Thirdly, the reaction intermediate (*i.e.*, *O*-acylisourea) was generated using SMARTS^43^ mapping between the carboxylic acid and the corresponding catalyst (see ESI Fig. S2†), following the mechanism proposed by Chan *et al*.^44^ The formation of *O*-acylisourea is the rate-determining step for carbodiimide-catalyzed amide coupling reactions, so obtaining the information for *O*-acylisoureas is theoretically beneficial for modeling the reaction yield.

### Calculating molecular descriptors

**Fig. 7:**
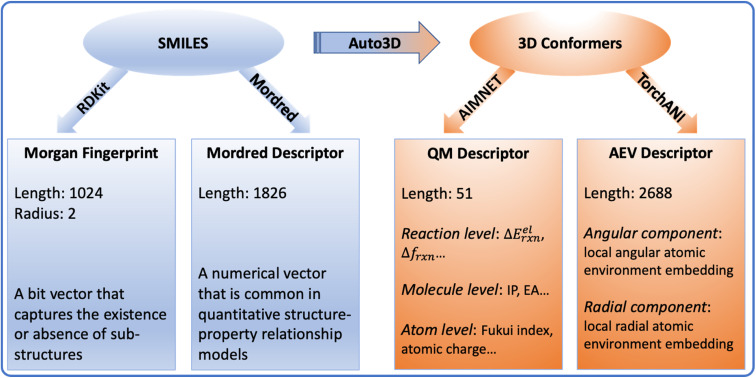
An overview of the molecular descriptors. Auto3D,^20^ RDKit,^45^ Mordred,^46^ AIMNET^22^ and TorchANI^47^ were used to transform molecules and calculate descriptors. Δ*E*^el^_rxn_ is the electronic energy difference between the product and reactants. Δ*f*_rxn_ is the Fukui index difference between acid and amine. IP is the ionization potential. EA is the electron affinity. AEV stands for “atomic environment vector”, which captures the local environment of an atom based on its neighbors in 3D space.

2D and 3D molecular descriptors were generated from the SMILES and conformers (Fig. 7). The 2D descriptors include Morgan fingerprint and Mordred descriptors, which are generated using RDKit^45^ and Mordred package,^46^ respectively. They are derived from reactant and product SMILES. Based on the optimized conformers, the 3D descriptors include the QM descriptor and the AEV descriptor. The AIMNET model^22^ was used to generate QM descriptors for reactants, products and *O*-acylisoureas, on which we derived additional quantities that capture reaction characteristics, such as the electronic reaction energy, charges and Fukui indices. The complete list of the original AIMNET descriptors and derived properties are available in Tables S1 and S2.† TorchANI was used to calculate the AEV descriptor,^47^ which captures the radial and angular distribution of the atomic environment. Since each AEV represents one atom of a molecule, we take the summation of atom AEVs to get the molecular representation. Due to the sparsity or missing values in Mordred and AEV descriptors, we filtered all features based on their variance thresholds as detailed in the ESI.† The surrogate models utilized for optimizing conformers and calculating descriptors approximate DFT-level accuracy *via* neural network implementation, ensuring the efficiency for large-scale applications.^48,49^ The accuracy and applicability of these models have undergone rigorous benchmarking in their respective source publications.^20,22,50^ For the benchmarking experiments, the choice of molecular representation was subject to the model category. The reaction graph is a concatenation of molecule graphs that are derived from SMILES. For numerical descriptors, the reaction vector is a concatenation of individual molecular descriptors.

### The models for benchmarking

A wide range of ML models were benchmarked on the amide coupling dataset, including linear methods, kernel methods, ensemble methods and neural networks. Specifically, the linear methods include linear regression with ridge or lasso regularization. The kernel methods include support vector machine (SVM). The ensemble methods include random forest (RF) and gradient boosting machine (GBM). We used the Scikit-Learn^51^ implementation for the above models. We included 3 types of graph neural networks (GNNs): NNConv,^24^ MFConv^25^ and AttentiveFP.^26^ They all achieved state-of-the-art results in some QSPR benchmarks. We used the PyTorch Geometric^52^ implementation of the above models. Yield-BERT^10^ has been shown to be successful to predict the yields on several datasets, so it is also included as one of the benchmark models here. The Yield-BERT model is available on the website.^53^ All other neural networks were implemented with PyTorch.^54^

The model performance was evaluated using 5 fixed and different train-test splits. For each train-test split, 90% of the reactions were used for training and the remaining reactions were used for testing. The hyperparameter searching was implemented with the sweep utility of WandB.^55^ In addition, the Buchwald–Hartwig dataset^9^ was used as a control dataset for all yield prediction models to provide an intuitive comparison between different methods. It is an HTE dataset that has been extensively used in many reaction yield prediction projects.^13,27,56^

## Data availability

The reaction data is under the patent of Reaxys. Links and IDs of the reactions, 3D molecular structures, and descriptors are available at: https://github.com/isayevlab/amide_reaction_data.

## Author contributions

Z. L. and O. I. conceptualized the project, Z. L. curated the dataset and built the models, O. I. supervised the project, and Z. L., Y. M. and O. I. contributed to data analysis and writing of the manuscript.

## Conflicts of interest

The authors declare no conflicts.

## Supplementary Material

SC-014-D3SC03902A-s001

SC-014-D3SC03902A-s002
