# Supplementary material for: The challenge of balancing model sensitivity and robustness in predicting yields: a benchmarking study of amide coupling reactions
Source: Chem Sci. 2023 Sep 13;14(39):10835–46. doi: 10.1039/d3sc03902a (PMC10566507; doi:10.1039/d3sc03902a)
Supplement: SC-014-D3SC03902A-s001 [file SC-014-D3SC03902A-s001.pdf]

Electronic Supplementary Material (ESI) for Chemical Science.
This journal is © The Royal Society of Chemistry 2023

Supporting Information

The Challenge of Balancing Model Sensitivity and Robustness in Predicting Yields: A Benchmarking Study of Amide Coupling Reactions

Zhen Liu[1], Yurii S. Moroz[2,3,4], Olexandr Isayev[1*]

[1]Department of Chemistry, Mellon College of Science, Carnegie Mellon University, Pittsburgh, Pennsylvania 15213, USA

[2]*Enamine Ltd, Kyïv, 02660, Ukraine*

[3]*Chemspace LLC, Kyïv, 02094, Ukraine*

[4]*Taras Shevchenko National University of Kyïv, Kyïv, 01601, Ukraine*

[*] Correspondence: olexandr@olexandrisayev.com (O.I.)

Extraction and curation of amide coupling reactions

The dataset for amide coupling reactions was obtained by querying the Reaxys database using predefined reaction templates. These templates were constructed by initiating a "Quick search" within the Reaxys interface. Subsequently, a generic amide coupling reaction was drawn, and this template was then applied to formulate queries for the desired reactions.

The approach involved sketching reactions using the MarvinJS interface integrated into Reaxys, akin to the process one might undertake on a physical whiteboard. This interface offers a multitude of functionalities. For instance, it allows for the specification of "R" groups denoting various atom assemblies, mapping of atoms from reactants to products, and delineation of the catalyst by illustrating it above the reaction arrow. A comprehensive guide for fundamental operations can be accessed here.

The initial download yielded approximately 195,800 amide coupling reactions catalyzed by carbodiimides. Subsequent refinement and curation procedures resulted in a final set of 41,239 reactions. For the sake of reproducibility, the Reaxys IDs corresponding to the reactions in our dataset can be found on the GitHub page: https://github.com/isayevlab/amide_reaction_data.

Reaction mechanism

The amide coupling dataset involves 3 types of carbodiimides: EDC, DIC and DCC (**Figure S1**). O-acylisourea is the key intermediate during the formation of amide. It has two pathways to form the desired amide. It also undergoes a slow rearrangement, producing the side product N-acylurea.

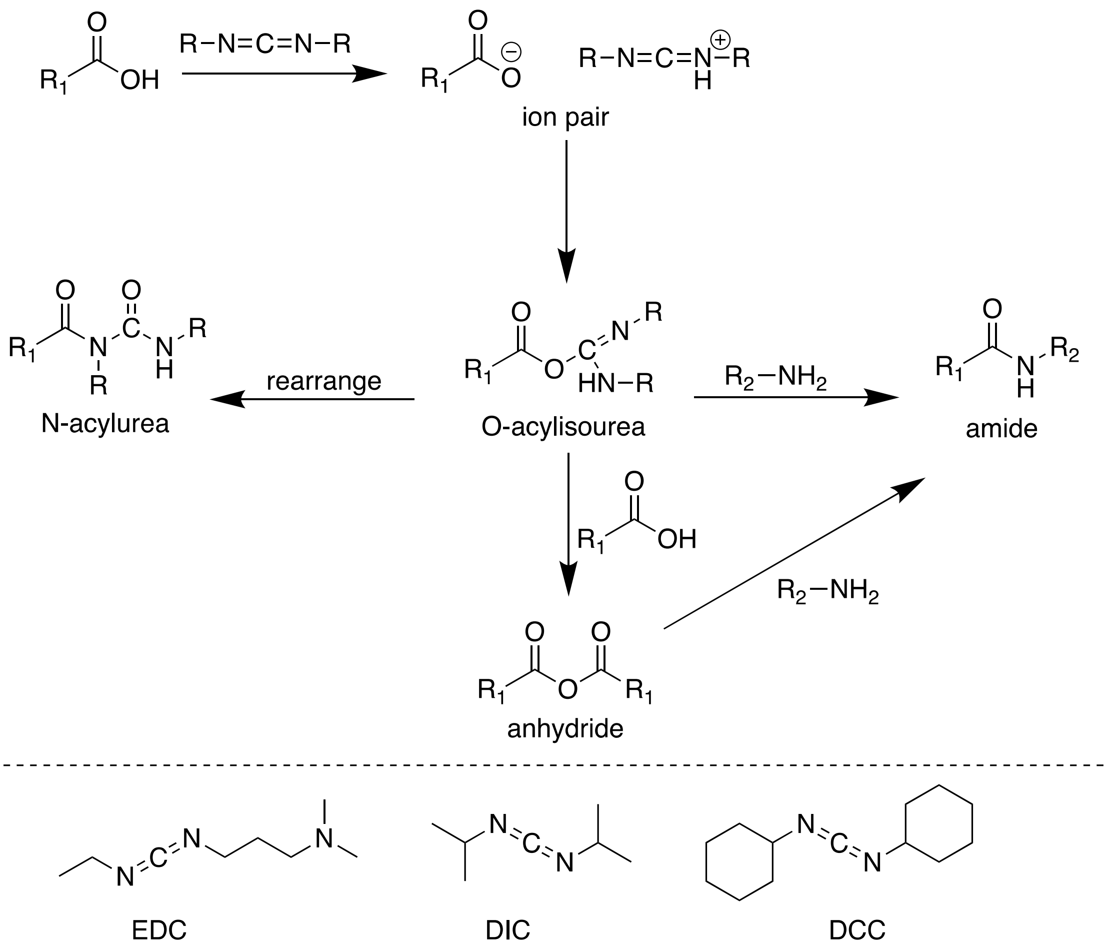

Figure S1. The mechanism for amide coupling catalyzed by carbodiimides.

Generation of O-acylisourea

All reactions in the amide coupling dataset follow the same mechanism, so the intermediates can be obtained via SMARTS pattern mapping. Below is an example of transforming 10 carboxylic acids into 10 O-acylisoureas by the reaction between acids and EDCI (**Figure S2**).

In [1]:
```
from rdkit import Chem
from rdkit.Chem import rdChemReactions
from rdkit.Chem import Draw
import pandas as pd

def acylisourea(smiles):
    rxn = rdChemReactions.ReactionFromSmarts('[C:1](=[O:2])[OH].CCN=C=NCCCN(C)C>>CCN/C([O][C:1]=[O:2])=N\CCCN(C)C')
    reacts = (Chem.MolFromSmiles(smiles), Chem.MolFromSmiles('CCN=C=NCCCN(C)C'))
    products = rxn.RunReactants(reacts)
    return Chem.MolToSmiles(products[0][0])
```

In [3]:
```
# get 10 example acids
df = pd.read_csv('/home/zhen/Documents/data/datav4/data4_4.csv')

acids = []
for i in range(8):
    acids.append(df.iloc[i, 12])
```

In [4]:
```
# Initial acid molecules
acid_mols = []
for smi in acids:
    acid_mols.append(Chem.MolFromSmiles(smi))

Draw.MolsToGridImage(acid_mols, molsPerRow=4)
```

Out[4]:

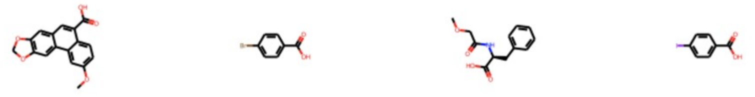

Figure S2. Example of generating O-acylisourea from acid and EDCI through SMARTS mapping

Annotation of the reactive centers

Algorithm 1 describes the process for identifying the reactive centers of the acids, amines and products. The isomorphic test was implemented via NetworkX[1]. It returns True if two groups of atoms have the same connectivity. For each molecule, the reactive center is the indexes of the reactive atoms in the molecular SDF format.

Algorithm 1. Amide Reaction Center Detector

Input: *acid, amine, product*
Output: reaction centers (*center*)

for every C-N *bond* in the *target*, **do**
    Break the product at the *bond*
    Re-construct *pseudo-acid* and/or *pseudo-amine* from the *fragment(s)*
    Isomorphic test between *pseudo-acid* and *acid*, *pseudo-amine* and *amine*
    if both tests are true, **then**
        return *product-centers*, *pseudo-acid*, *pseudo-amine*
    end if
end for

for $atom_i$ in the *acid*/*amine*, **do**
    Find the neighboring graph around $atom_i$ (G_i)
    for $atom_j$ in the *fragment-acid/fragment-amine*, **do**
        Find the neighboring graph around $atom_j$ (G_j)
        Isomorphic test on G_i and G_j
    end for
    if no $atom_j$ that has the same neighboring graph as $atom_i$, **then**
        return *acid/amine-center*
    end if
end for

Generation of QM descriptors

With the 3D structures from Auto3D[2], the descriptors from **Table S1** can be calculated directly from AIMNET[3]. From these original descriptors, we derived 51 descriptors that capture the properties of the amide reactions in **Table S2**.

Table S1. Original AIMNET descriptors		
**Descriptor**	**Data Type and Shape**	**Explanation**
energy	list, length=3	The energies for three states (+1, 0, -1) of the input molecule, unit eV
energy_std	list, length=3	Standard deviation from the AIMNET ensemble (5 models in total)
charges	2D list, (3, number of atoms)	The atomic charges (summation of the alpha and beta charges) for three states (+1, 0, -1) of the input molecule
charges_std	3D list, (3, number of atoms, 2)	Standard deviation of the atomic alpha and beta charge from the AIMNET ensemble
ip	float	ionization potential, unit eV
ea	float	electron affinity, unit eV
f_el	list, length=number of atoms (including H)	atomic Fukui functions for electronic attack
f_nuc	list, length=number of atoms (including H)	atomic Fukui function for nucleophilic attack
f_rad	list, length=number of atoms (including H)	atomic Fukui function for radical attack
chi	float	electronegativity, unit eV
eta	float	hardness, unit eV
omega	float	electrophilicity index, unit eV
omega_el	list, length=number of atoms (including H)	atomic electrophilicity index for electrophilic attack, unit eV
omega_nuc	list, length=number of atoms (including H)	atomic electrophilicity index for nucleophilic attack, unit eV
omega_rad	list, length=number of atoms (including H)	atomic electrophilicity index for radical attack, unit eV

Table S2. The QM descriptors for amide coupling reactions		
**Descriptor**	**Explanation**	**Formula**
ΔE_{rxn}^{el}	electronic reaction energy	$\Delta E_{rxn}^{el} = E_{product}^{el} + E_{water}^{el} - E_{acid}^{el} - E_{amine}^{el}$
ΔE_{i}^{el}	the electronic energy difference between the intermediate and the reactant	$\Delta E_{i}^{el} = E_{intermediate}^{el} - E_{acid}^{el} - E_{catalyst}^{el}$

rxn_acid_fukui	Fukui index	$f^{nuc}_{amine\ center} - f^{rad}_{amine\ center}$
rxn_amine_fukui	Fukui index	$f^{el}_{acid\ center} - f^{nuc}_{acid\ center}$
rxn_fukui	Fukui index	$f_{rxn} = f^{nuc}_{amine\ center} - f^{rad}_{amine\ center}) - (f^{el}_{acid\ center} -$
acid_ip	ionization energy potential	IP_{acid}
amine_ip	ionization energy potential	IP_{amine}
p_ip	ionization energy potential	$IP_{product}$
aa_ip	ionization energy potential	$IP_{amine} - IP_{acid}$
rxn_ip	ionization energy potential	$IP_{rxn} = IP_{product} - IP_{acid} - IP_{amine}$
acid_ea	electron affinity	EA_{acid}
amine_ea	electron affinity	EA_{amine}
p_ea	electron affinity	$EA_{product}$
aa_ea	electron affinity	$EA_{amine} - EA_{acid}$
rxn_ea	electron affinity	$EA_{rxn} = EA_{product} - EA_{acid} - EA_{amine}$
acid_chi	electronegativity	χ_{acid}
amine_chi	electronegativity	χ_{amine}
p_chi	electronegativity	$\chi_{product}$
aa_chi	electronegativity	$\chi_{amine} - \chi_{acid}$
rxn_chi	electronegativity	$\chi_{rxn} = \chi_{product} - \chi_{acid} - \chi_{amine}$
acid_eta	hardness	η_{acid}
amine_eta	hardness	η_{amine}
p_eta	hardness	$\eta_{product}$
aa_eta	hardness	$\eta_{amine} - \eta_{acid}$
rxn_eta	hardness	$\eta_{rxn} = \eta_{product} - \eta_{acid} - \eta_{amine}$
acid_omega	electrophilicity index	ω_{acid}
amine_omega	electrophilicity index	ω_{amine}
p_omega	electrophilicity index	$\omega_{product}$
aa_omega	electrophilicity index	$\omega_{amine} - \omega_{acid}$
rxn_omega	electrophilicity index	$\omega_{rxn} = \omega_{product} - \omega_{acid} - \omega_{amine}$
C_charge	atomic charge on the acid carbon center	
N_charge	atomic charge on the amine nitrogen center	

pC_charge	atomic charge on the product carbon center
pN_charge	atomic charge on the product nitrogen center
pCC_charge	pCC_charge = pC_charge - C_charge
pNN_charge	pNN_charge = pN_charge - N_charge
CN_charge	CN_charge = N_charge - C_charge
C_fukui	Fukui index on the acid carbon center
N_ fukui	Fukui index on the amine nitrogen center
pC_ fukui	Fukui index on the product carbon center
pN_ fukui	Fukui index on the product nitrogen center
pCC_fukui	pCC_fukui = pC_fukui - C_fukui
pNN_fukui	pNN_fukui = pN_fukui - N_fukui
CN_fukui	CN_fukui = N_fukui - C_fukui
C_omega	electrophilicity index on the acid carbon center
N_omega	electrophilicity index on the amine nitrogen center
pC_omega	electrophilicity index on the product carbon center
pN_omega	electrophilicity index on the product nitrogen center
pCC_omega	pCC_omega = pC_omega - C_omega
pNN_omega	pNN_omega = pN_omega - N_omega
CN_omega	CN_omega = N_omega - C_omega

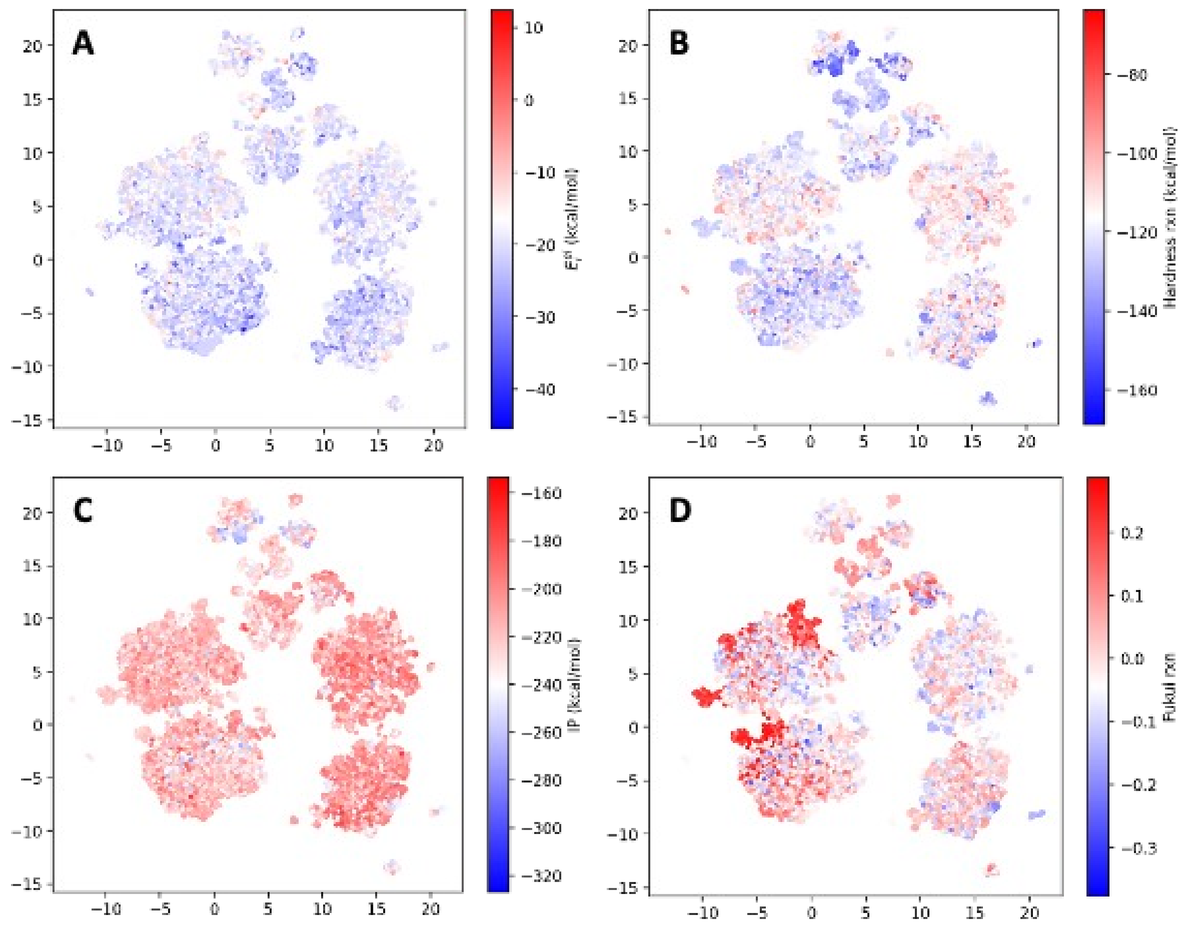

Figure S3. UMAP projection of the reaction space colored by additional QM features.

Generation of AEV and Mordred descriptors

The atomic environment vector (AEV) descriptor was calculated using the AEV computer in TorchANI[4]. We considered 12 elements for the AEV calculator to cover all the molecules in the amide coupling dataset. The conversion rule from the atomic number to the atomic index in TorchANI was {1:0, 5: 1, 6: 2, 7: 3, 8: 4, 9: 5, 14: 6, 15: 7, 16: 8, 17: 9, 35: 10, 53: 11}. The other parameters for the AEV computer are default values. The resulting AEV has a dimension of 2,688. Since many columns contain mostly the same values, we only kept the AEV features of which the variance is at least 0.0001. The final AEV feature length is 1,716.

The Mordred descriptor was calculated using the Mordred package[5]. The original Mordred descriptor contains 1,826 features. After removing features with missing values, we ended up with 1648 features.

Amide coupling dataset statistics

After downloading from Reaxys, reactions involving organometallic compounds or very large molecules were excluded. After processing, each reactant contains no more than 100 atoms, and each product contains no more than 150 atoms. The raw dataset contained approximately 195,800 amide coupling reactions with different catalysts, from which we sampled a subset of reactions that are catalyzed by carbodiimides. The carbodiimides include N,N'-Dicyclohexylcarbodiimide (DCC), 1-Ethyl-3-(3-dimethylaminopropyl)carbodiimide (EDC) and N,N′-Diisopropylcarbodiimide (DIC).

The amide coupling dataset contains 41,239 reactions, which consist of 111,071 unique molecules. There are hundreds to thousands of low-yield reactions, although the yield distribution is left-skewed (i.e., most reactions have high yields). Most reactions involve medium to large molecules because the product molecular weight is between 250 to 650. More statistics about the dataset can be found in **Figure S4 and S5**.

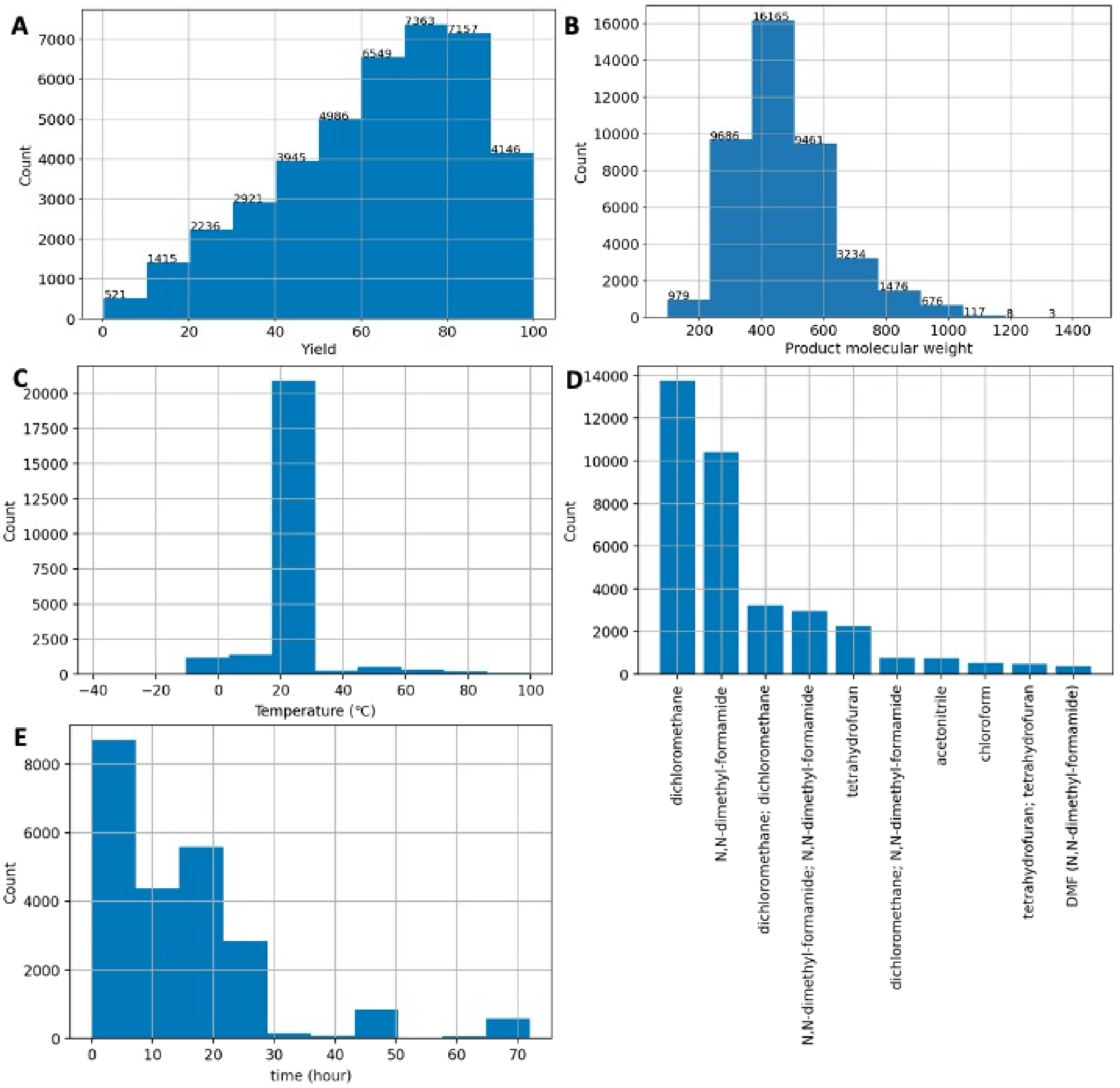

Figure S4. The distribution of reaction yield (A), size (B), temperature (C), solvent (D) and time (E).

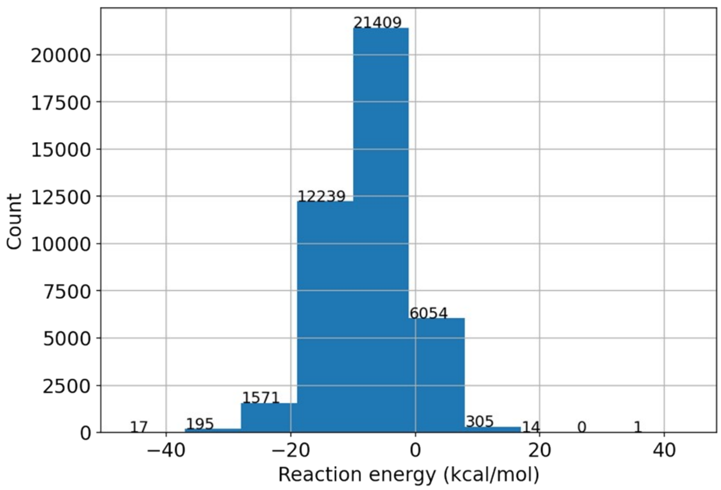

Figure S5. The distribution of electronic reaction energy

The MAE metric on the amide coupling dataset and the Buchwald-Hartwig dataset

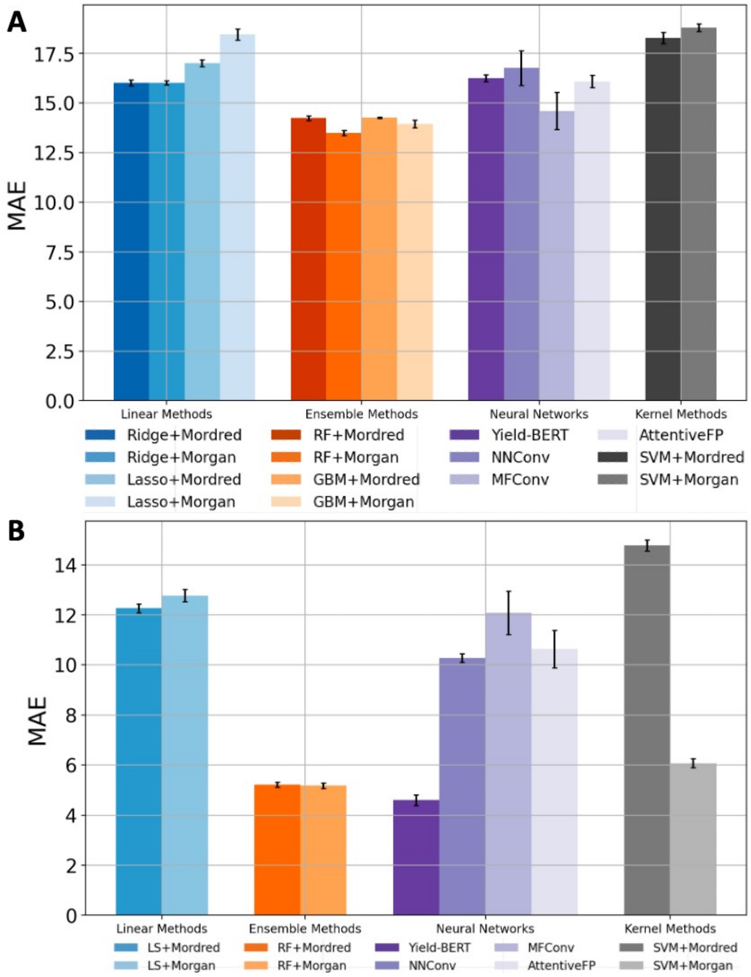

Figure S6. The MAE metric for yield prediction on the amide coupling dataset (A) and Buchwald-Hartwig dataset (B).

Recursive feature elimination (RFE)

RFE is a technique for selecting the most informative features. The process is as follows: a model is first trained using the full features. The features are then ranked by the importance assigned by this trained model. A subset of the features will be pruned based on the importance ranking. The remaining features are used to train another model, then we further prune the features based on the new importance scores… This process is repeated until the total number of features decreases to a specific threshold. In our case, we trained an RF at each step and used it for feature

selection. At each step, we removed the bottom 20% of the features based on the importance ranking. **Figure S7** summarizes the model performance along the feature elimination process.

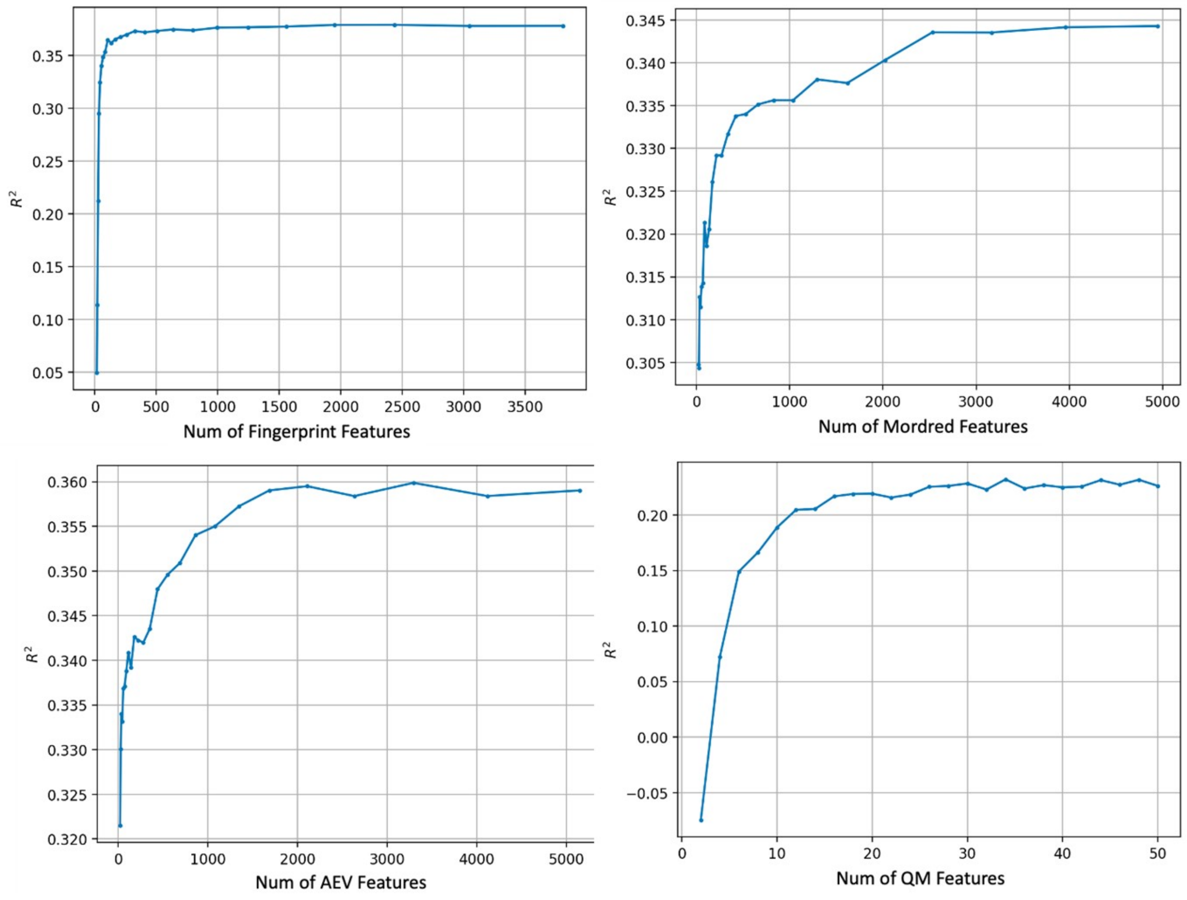

Figure S7. The model performance during the feature elimination process.

Figure S8 displays the top-10 most important features for each descriptor as identified during the Recursive Feature Elimination (RFE) process. The importance on the x-axis is derived from the "feature_importance_" attribute of a trained random forest, computed based on the mean decrease in impurity within each tree.

In Panel A, the y-axis portrays the substructure or condition linked with each bit of the fingerprint. In Panel B, the y-axis elucidates the physical significance of individual features within the Mordred descriptor. Panel C's y-axis signifies the coordinates of the top-10 features of the AEV, although the AEV descriptor lacks a corresponding physical interpretation. Meanwhile, in Panel D, the y-axis represents the QM terms, as outlined in **Table S2**.

It is worth noting that despite the heightened importance of the QM terms in comparison to other features, relying solely on the QM descriptor did not yield satisfactory performance.

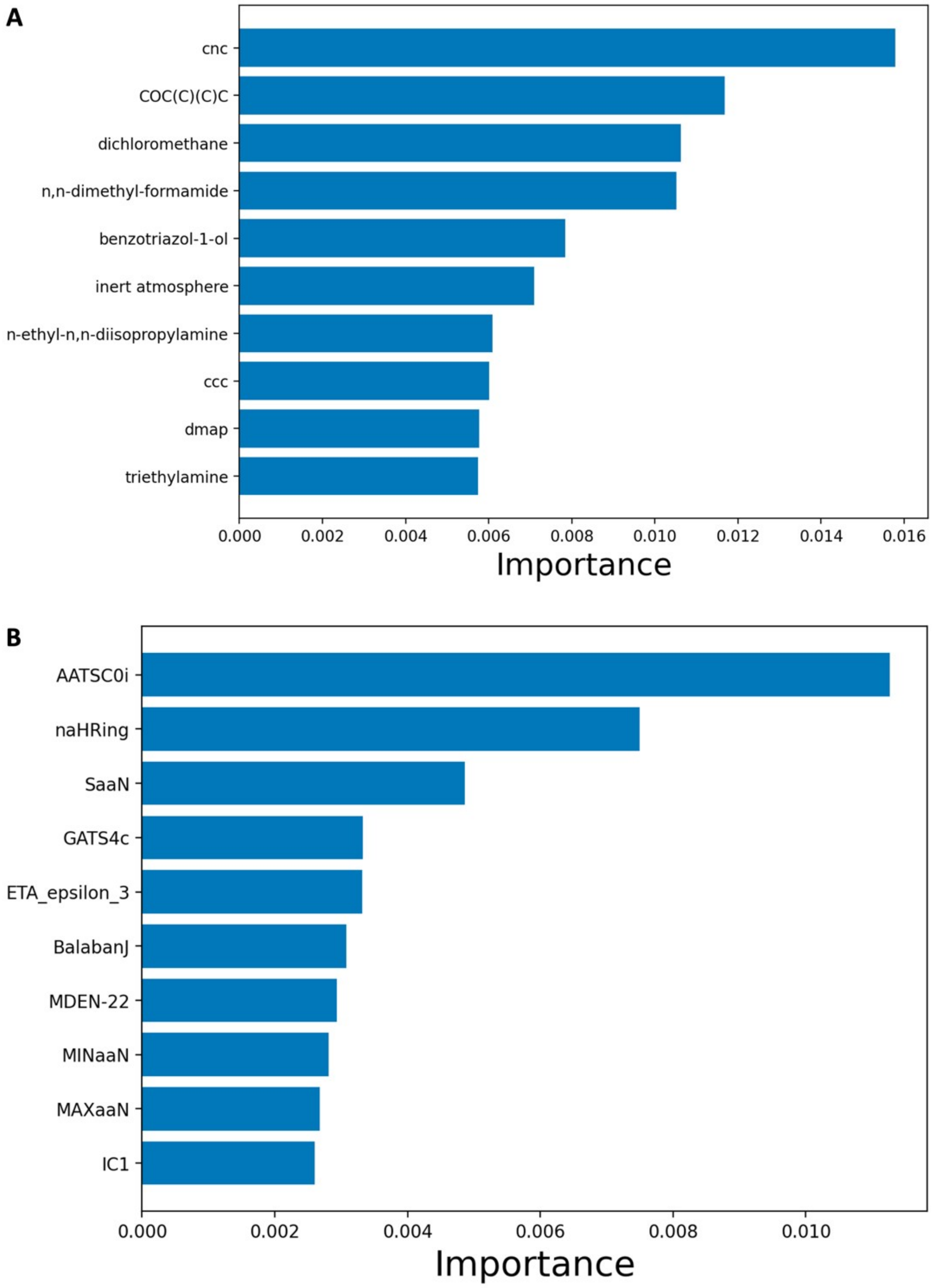

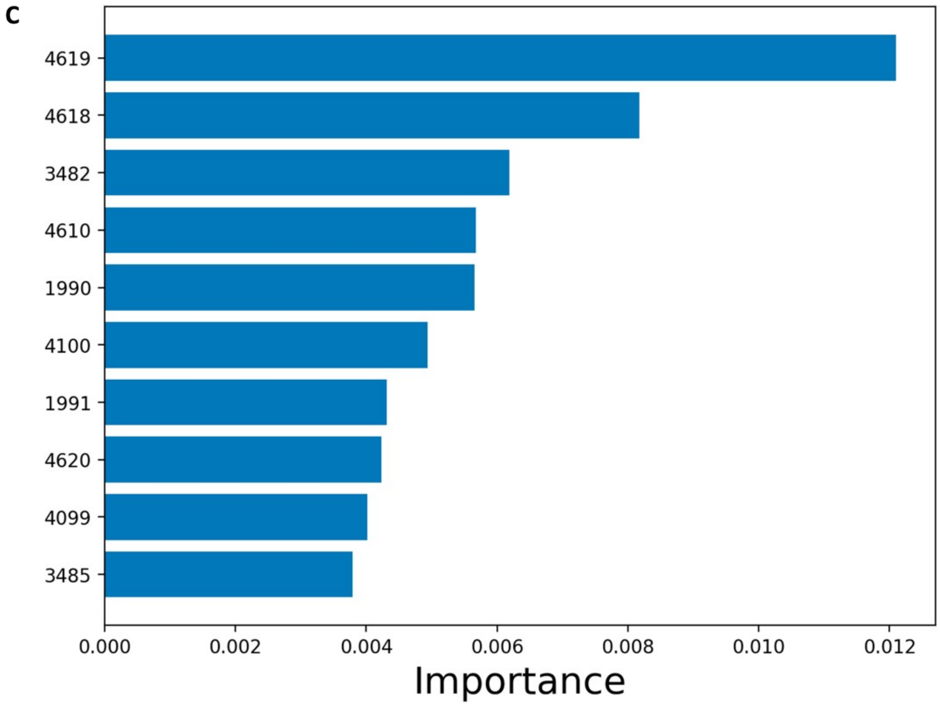

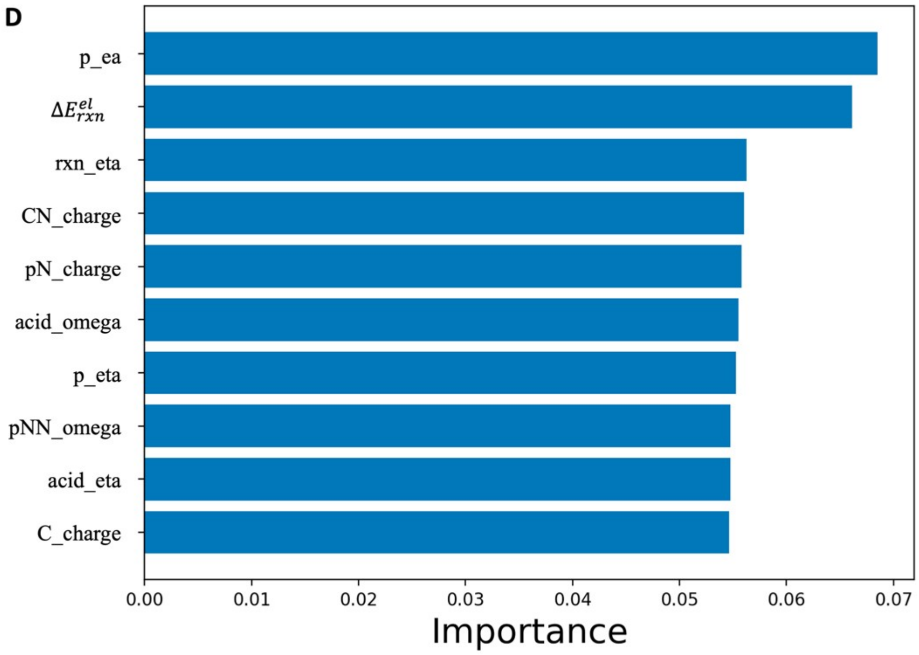

Figure S8. The top-10 most important features in the fingerprint descriptor (A), Mordred descriptor (B), AEV descriptor (C) and the QM descriptor (D).

Yield prediction performance on different amine types

For each type of amine, 90% of the data was allocated for training purposes, while the remaining 10% was reserved for testing. The identical stacking technique and descriptors, as discussed in the main text, were employed in this context as well.

Table S3. Yield prediction performance on different subsets of the amide coupling dataset

Amine Category	primary aliphatic	primary aromatic	secondary
**Subset Size**	28,067	12,530	634
**R^2**	0.363	0.425	0.424
**MAE (%)**	13.29	13.42	14.29

Model performance after injecting artificial negative reactions

The following experiments were done using reaction context and fingerprint as the descriptor. The artificial negative reactions were produced by changing the fingerprint bits for the acid or amine to be all zeros and then assigning the reaction yield as 0. The logic behind these artificial negative data points was that the reaction cannot happen when either acid or amine was missing.

Table S4. Model performance with artificial negative data augmentation

Model	Percent of negative data points in the training set	R^2	MAE (%)
RF	0	0.348	13.97
	20	0.341	14.07
	50	0.340	14.07
	100	0.342	14.06
MLP	0	0.331	14.03
	20	0.331	13.91
	50	0.312	13.98
	100	0.312	13.98

Comparison between the HTE dataset and the amide dataset

The details of the HTE dataset was discussed by Doyle et al[6]. We added the side-by-side comparison of the HTE dataset and the amide dataset curated in this work. Compared with the HTE dataset, the amide dataset is around 10 times larger in terms of reaction size, around 1500 times larger in terms of unique molecules, and contains comprehensive (though not complete) information about molecules and reactions. Besides the difference in dataset size, a notable difference is the yield distribution. For the HTE dataset, a larger portion of the reactions have low yields, but a large portion of reactions have high yields for the amide dataset. It is commonly

believed that lacking negative reaction is a major reason for the poor performance on large literature dataset. However, the R^2 can be as low as 0.2 even on a large electronic notebook dataset where there is a large portion of negative examples[7]. This phenomenon indicates the existence of other factors that degrading model performance in yield prediction. Compared with other literature dataset, a unique advantage of our amide dataset is that all reactions follow the same mechanism. We could identify many similar reactions and observe how subtle structure change could lead to the difference in the final yield, providing the opportunity to observe the reactivity cliffs and uncertain yields.

**Table S5. Comparing the HTE dataset and the amide dataset**		
**Metric**	**HTE dataset**	**Amide dataset**
Number of reactions	4,608	41,239
Number of unique reactants	45 (15 aryl halides, 23 additives, 4 Pd catalysts, 3 bases)	70,081 (16,285 acids, 12,598 amines, 3 carbodiimide catalysts, 17,518 intermediates)
3D structure	NO	Yes
Intermediate	NO	Yes
Context	NO	Yes
Yield distribution	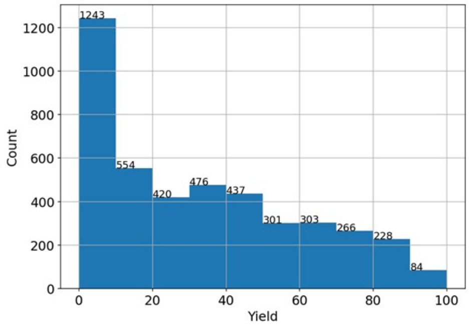	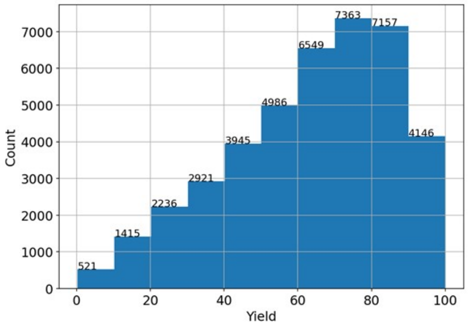

Reference

(1) Hagberg, A. A.; Schult, D. A.; Swart, P. J. Exploring Network Structure, Dynamics, and Function Using NetworkX. In *7th Python in Science Conference (SciPy 2008)*; 2008; pp 11–15.

(2) Liu, Z.; Zubatiuk, T.; Roitberg, A.; Isayev, O. Auto3D: Automatic Generation of the Low-Energy 3D Structures with ANI Neural Network Potentials. *J. Chem. Inf. Model.* **2022**, *62*, 5373–5382.

(3) Zubatyuk, R.; Smith, J. S.; Nebgen, B. T.; Tretiak, S.; Isayev, O. Teaching a Neural Network to Attach and Detach Electrons from Molecules. *Nat. Commun.* **2021**, *12*, 1–11.

(4) Gao, X.; Ramezanghorbani, F.; Isayev, O.; Smith, J. S.; Roitberg, A. E. TorchANI: A Free and Open Source PyTorch-Based Deep Learning Implementation of the ANI Neural Network Potentials. *J. Chem. Inf. Model.* **2020**, *60*, 3408–3415.

(5) Moriwaki, H.; Tian, Y. S.; Kawashita, N.; Takagi, T. Mordred: A Molecular Descriptor Calculator. *J. Cheminform.* **2018**, *10*, 14.

(6) Ahneman, D. T.; Estrada, J. G.; Lin, S.; Dreher, S. D.; Doyle, A. G. Predicting Reaction Performance in C–N Cross-Coupling Using Machine Learning. *Science (80-.).* **2018**, *360*, 186–190.

(7) Wiest, O.; Saebi, M.; Nan, B.; Herr, J. E.; Wahlers, J.; Guo, Z.; Zuranski, A.; Kogej, T.; Norrby, P.-O.; Doyle, A. G.; Chawla, N. V. On the Use of Real-World Datasets for Reaction Yield Prediction. *Chem. Sci.* **2023**, *14*, 4997–5005.
