# Supplementary material for: The challenge of balancing model sensitivity and robustness in predicting yields: a benchmarking study of amide coupling reactions
Source: Chem Sci. 2023 Sep 13;14(39):10835–46. doi: 10.1039/d3sc03902a (PMC10566507; doi:10.1039/d3sc03902a)
Supplement: SC-014-D3SC03902A-s002 [file SC-014-D3SC03902A-s002.pdf]

Electronic Supplementary Material (ESI) for Chemical Science.
This journal is © The Royal Society of Chemistry 2023

Supporting Information

The Challenge of Balancing Model Sensitivity and Robustness in Predicting Yields: A Benchmarking Study of Amide Coupling Reactions

Zhen Liu[1], Yurii S. Moroz[2,3,4], Olexandr Isayev[1*]

[1]Department of Chemistry, Mellon College of Science, Carnegie Mellon University, Pittsburgh, Pennsylvania 15213, USA

[2]*Enamine Ltd, Kyïv, 02660, Ukraine*

[3]*Chemspace LLC, Kyïv, 02094, Ukraine*

[4]*Taras Shevchenko National University of Kyïv, Kyïv, 01601, Ukraine*

[*] Correspondence: olexandr@olexandrisayev.com (O.I.)

Examples of reactivity cliffs

The similarity of reactions was determined using the reaction fingerprint, and two reactions were deemed similar if their cosine similarity exceeded 0.90. Reactions were considered "cliffs" when their similarity surpassed 0.9, yet the yield difference was greater than 30. In our amide coupling datasets, we identified 6,365 reactivity cliffs. These examples serve to illustrate that even a minor alteration in molecular structure can result in a substantial variation in reaction yield. This observation underscores that the yield prediction surface is non-smooth, highlighting the challenges for accurately predicting reaction yields.

Reaxys ID	**Reaction**	**Yield (%)**	**Condition**
37377749	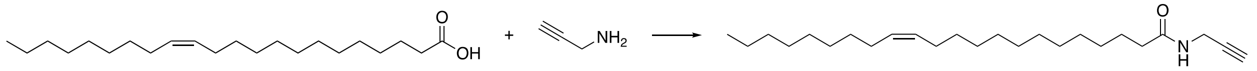	61	18 ℃, 20 h; DMAP, DIC, DCM
37377774	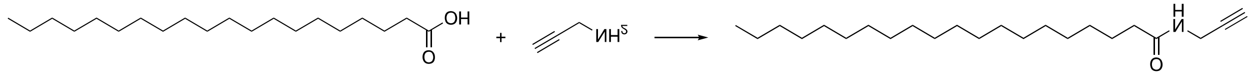	13	18 ℃, 20 h; DMAP, DIC, DCM
28563907	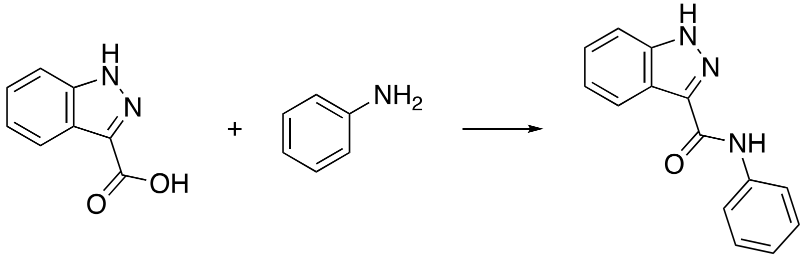	81	100 h; benzotriazol-1-ol, EDC, DMF
42264425	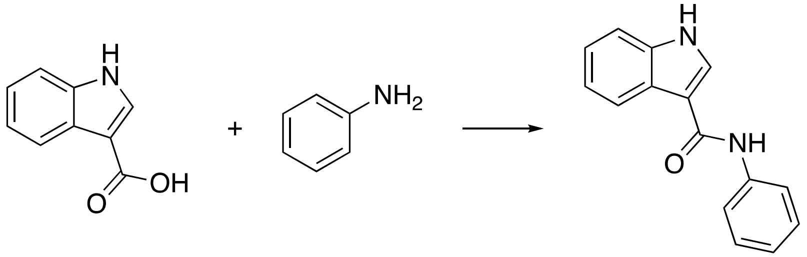	13	10 ℃, 100 h; benzotriazol-1-ol, EDC, DMF
28683797	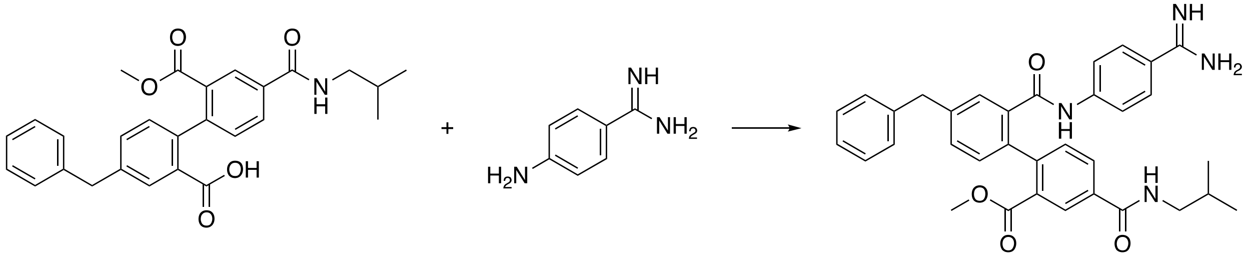	20	16.0 ℃, 20 h; Inert atmosphere, pyridine, DCC, DMF

28683737	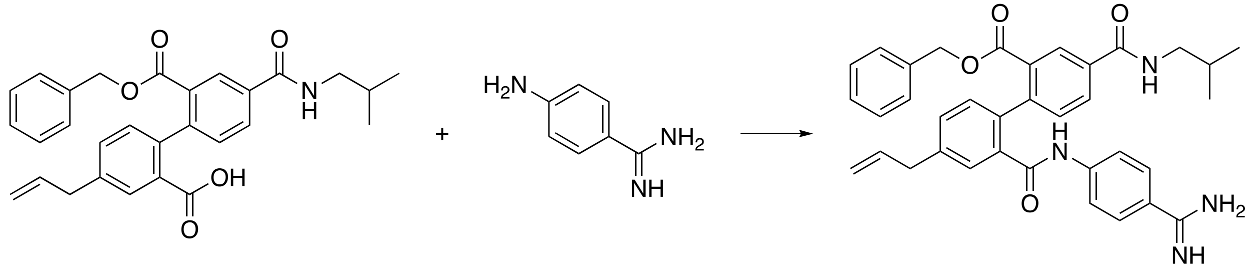	96	16 ℃, 20 h;<br>Inert atmosphere,<br>pyridine, DCC, DMF
51739779	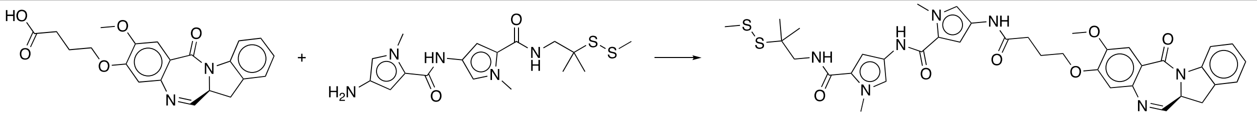	2	1 ℃, 25 h;<br>DMAP, EDC, DCM
52011231	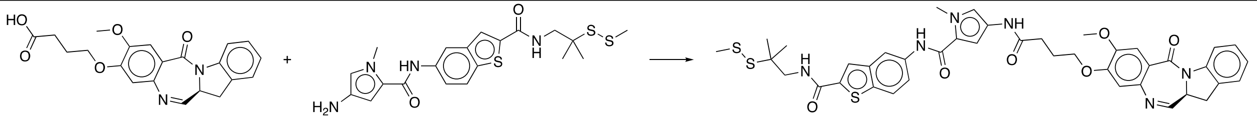	83	2 ℃, 20 h;<br>DMAP, EDC, DCM
42724738	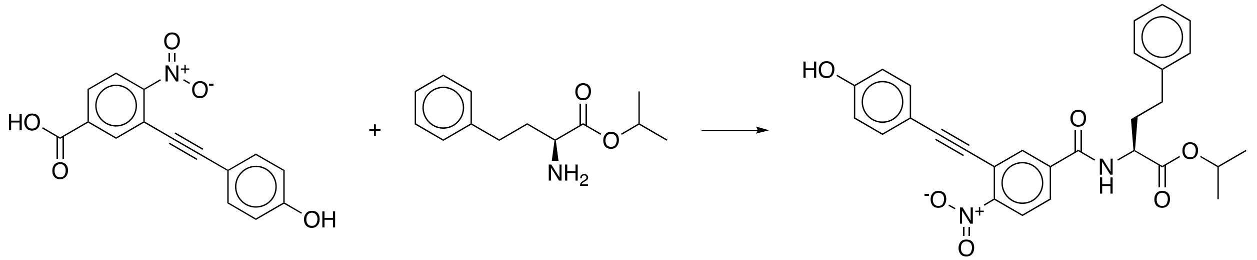	97	25 h;<br>2,6-dimethylpyridine;<br>1-hydroxy-7-aza-<br>benzotriazole;<br>EDC, DMF
42724744	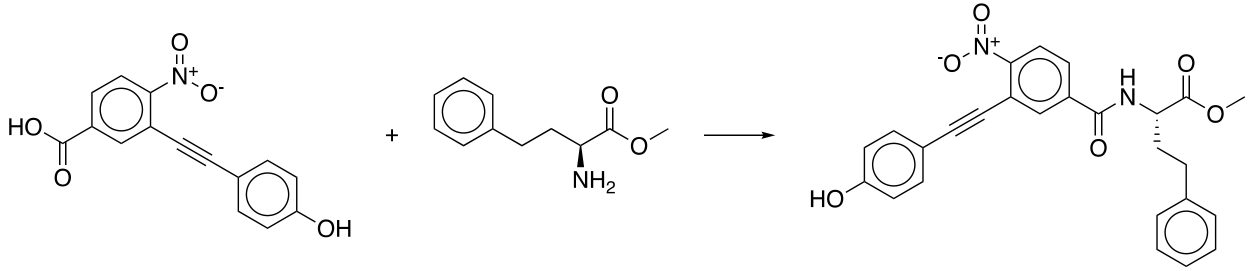	17	25 h;<br>2,6-dimethylpyridine;<br>1-hydroxy-7-aza-<br>benzotriazole;<br>EDC, DMF
36270730	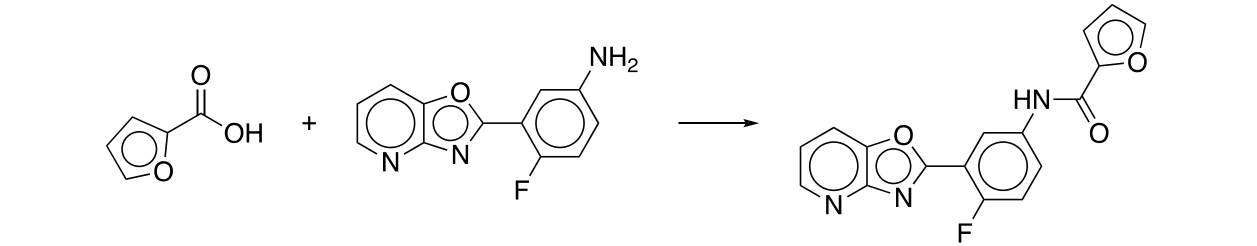	4	45 h;<br>Inert atmosphere, DMAP;<br>EDC, DCM

36270732	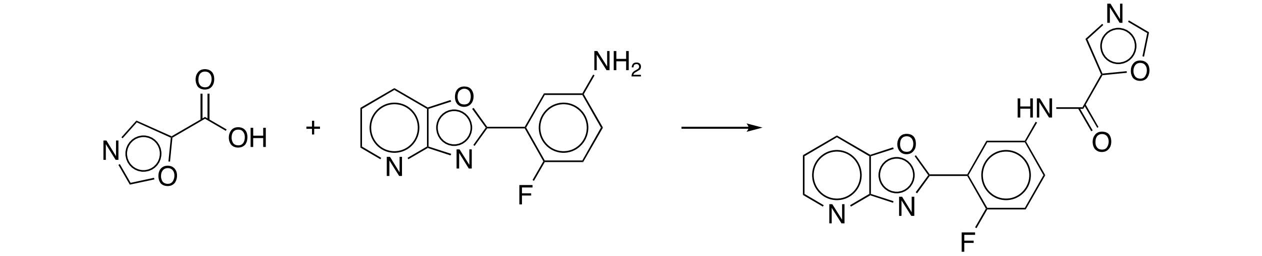	93	45 h;<br>Inert atmosphere, DMAP;<br>EDC, DCM
9190843	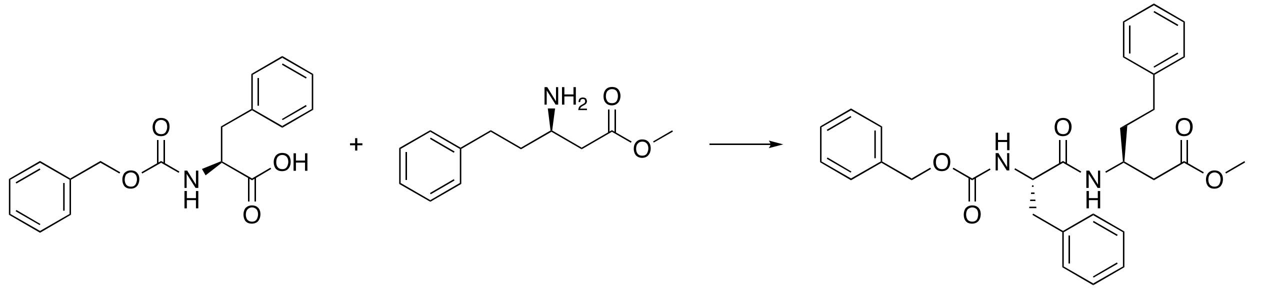	0.23	benzotriazol-1-ol;<br>EDC, DCM
2107062	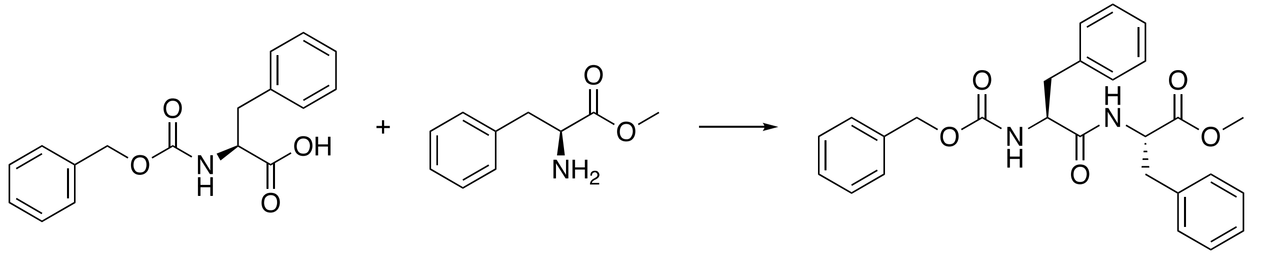	93	20 h;<br>4-methyl-morpholine;<br>benzotriazol-1-ol;<br>EDC, DMF
51739741	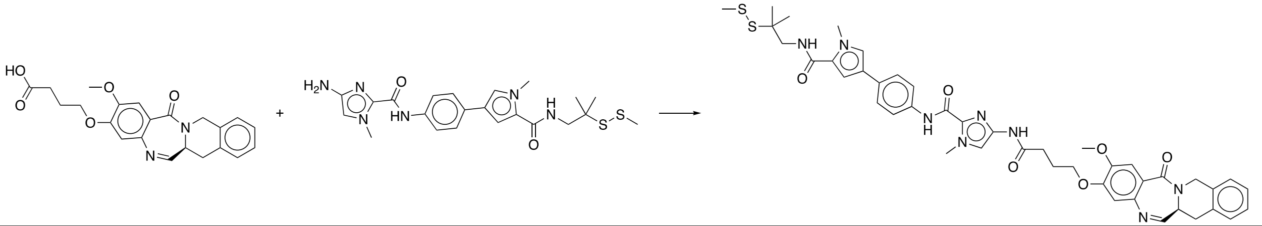	29	2 ℃, 25 h;<br>DMAP;<br>EDC, DCM
51739653	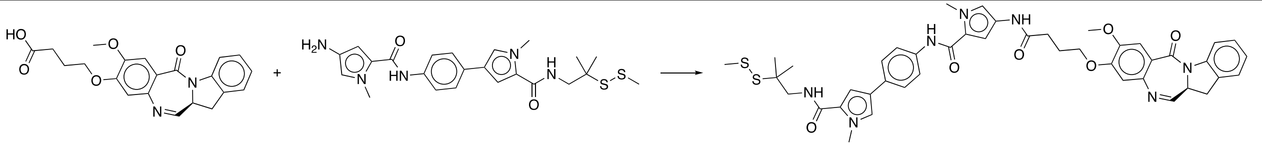	100	2 ℃, 20 h;<br>DMAP;<br>EDC, DCM

42264445	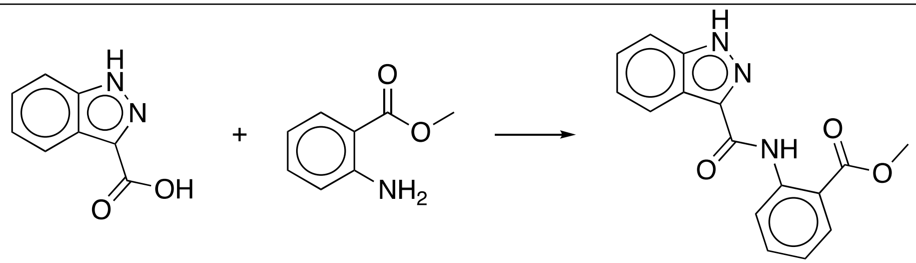	6	10 ℃, 100 h; benzotriazol-1-ol; EDC, DMF
42264442	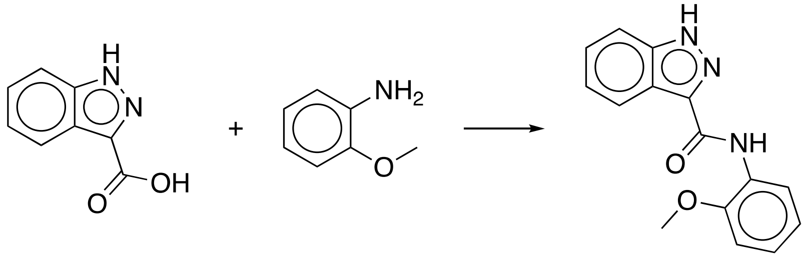	82	10 ℃, 100 h; benzotriazol-1-ol; EDC, DMF
5267967	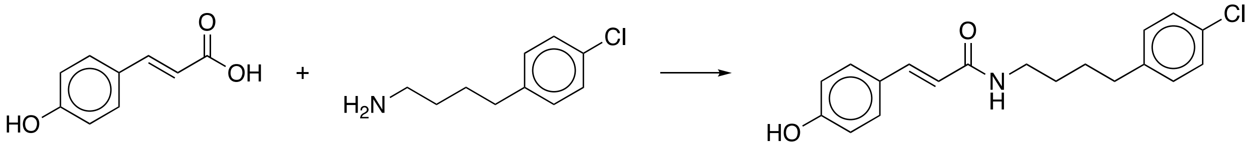	9	12 ℃, 80 h; benzotriazol-1-ol; DCC, DMF
4832409	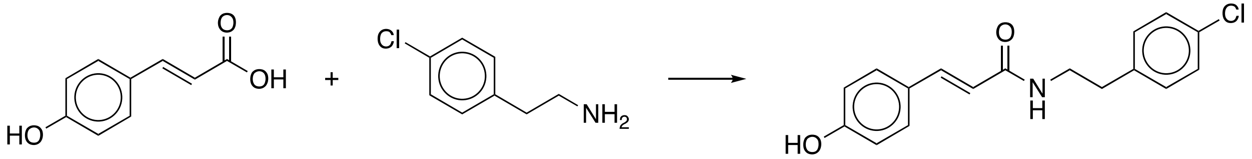	96	12 ℃, 80 h; benzotriazol-1-ol; DCC, DMF

Examples of uncertain reactions

The reactions were deemed to have "uncertain" yields when the carboxylic acid, amine, and product were the same, yet the yields (obtained from the Reaxys database) differed by at least 30. A group of uncertain reactions share the same catalyst, despite slight variations in the experimental context. Within our amide coupling dataset, we identified 649 reactions with uncertain yields. These instances emphasize that reproducibility poses an additional challenge when constructing accurate yield prediction models.

Reaxys ID	Reaction	Yield records in the Reaxys database (%)
712688	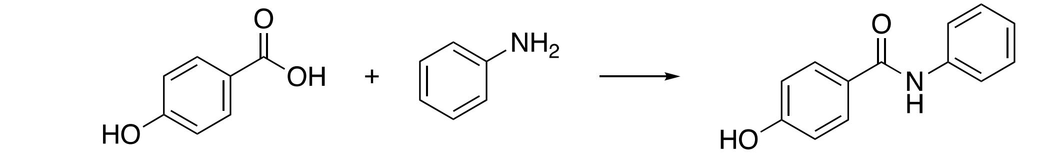	29, 71
1889311	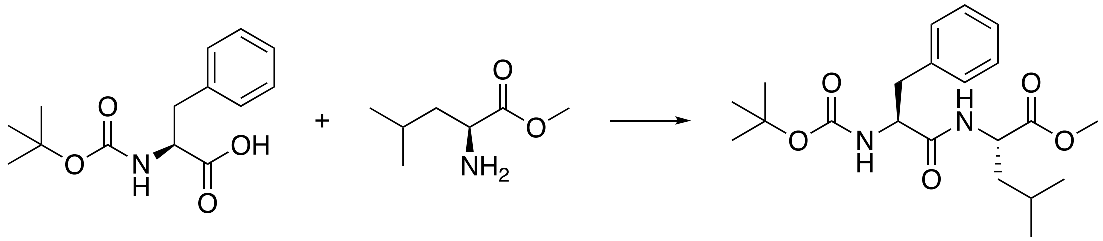	66, 70, 85, 87,
5211116	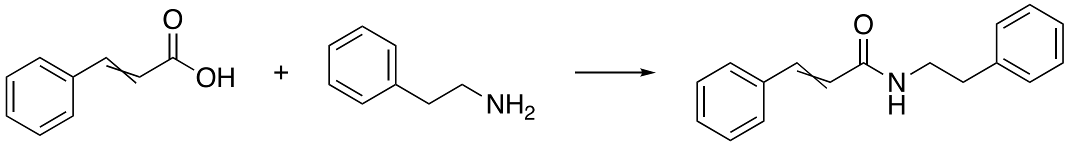	84, 96
44962717	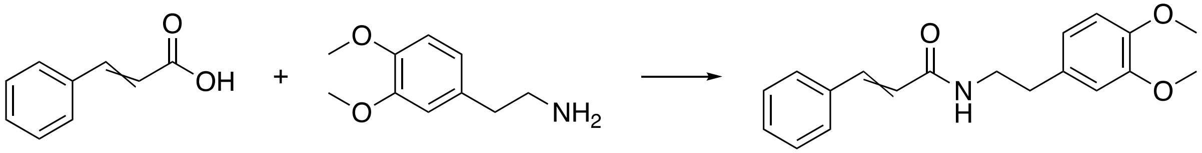	32.5, 97.5
28286573	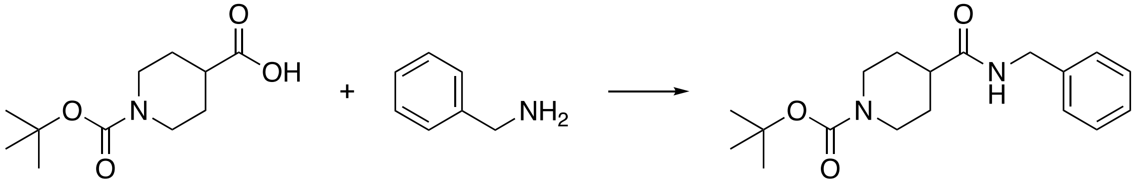	23, 62, 62, 99

1789336	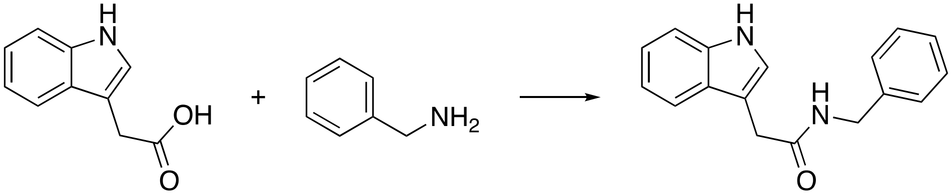	35, 85, 90
1820687	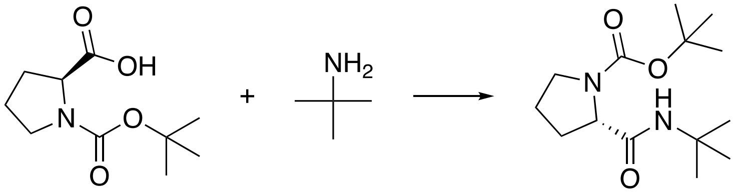	40, 40, 93
3747743	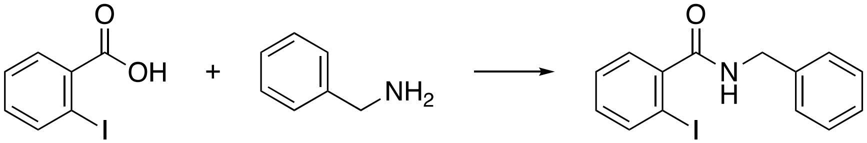	60, 60, 99
